# Competing Risk Prediction of COVID-19 Hospital Mortality with Model Diagnostics and Exploratory Evaluation in Mexico

**DOI:** 10.3390/diagnostics16203267

**Published:** 2026-10-09

**Authors:** Zahra Sabzevari, Hamid Reza Marateb, Reza Rasti, Martin Wolkewitz, Manuel Rubio-Rivas, Augusto Rojas-Martinez

**Affiliations:** 1 Biomedical Engineering Department, Engineering Faculty, University of Isfahan, Isfahan 81746-73441, Iran; z.sabzevari@eng.ui.ac.ir (Z.S.); r.rasti@eng.ui.ac.ir or reza.rasti@duke.edu (R.R.); 2 Institute for Research and Innovation in Health (IRIS), Automatic Control (ESAII), Universitat Politècnica de Catalunya (UPC), 08028 Barcelona, Spain; 3 Department of Biomedical Engineering, Duke University, Durham, NC 27708, USA; 4 Institute of Medical Biometry and Statistics, Faculty of Medicine and Medical Center, University of Freiburg, 79104 Freiburg, Germany; martin.wolkewitz@uniklinik-freiburg.de; 5 Department of Internal Medicine, Hospital Universitari General de Catalunya, Grupo Quirónsalud, Carrer de Pedro i Pons 1, 08190 Sant Cugat del Vallès, Spain; manuel.rrivas@quironsalud.es; 6 Tecnologico de Monterrey, Escuela de Medicina y Ciencias de la Salud, 64700 Monterrey, Mexico; augusto.rojasmtz@tec.mx

**Keywords:** COVID-19, prognosis, in-hospital mortality, competing risks, cause-specific Cox model, proportional hazards, nonlinear effects, calibration

## Abstract

**Background/Objectives:** We evaluated six-predictor cause-specific models for 45-day in-hospital COVID-19 mortality, including departures from proportional hazards and linearity and transport to Mexican data. **Methods:** The oxygenation-expanded Barcelona cohort included 1622 adults. Recorded arterial PaO_2_/FiO_2_ (P/F) was preferred; otherwise P/F was derived from documented saturation and inspired oxygen using the Rice relationship at SpO_2_ ≤ 97%. Linear cause-specific Cox models for death and discharge served as a reference. Quadratic and time-varying extensions were assessed by three repetitions of five-fold cross-validation. Frozen Barcelona models were evaluated in a separate Mexican workbook using reconstructed day-45 outcomes and minimum-oxygenation proxies. Historical supplied scores for 634 patients were analysed separately. **Results:** Barcelona included 325 day-45 deaths. The reference model had apparent area under the receiver operating characteristic curve (AUC) 0.822 and bootstrap-corrected AUC 0.820. Proportional hazards (PH) departures persisted within 45 days. Combining quadratic terms and predictor-by-log(1 + time) interactions improved cross-validated AUC from 0.819 to 0.831 and Brier score from 0.12343 to 0.11974, while calibration slope decreased from 0.945 to 0.830. In 171 Mexican records with 12 reconstructed deaths, AUC was 0.967 for the expanded linear model and 0.857 for the combined extension. Excluding one extreme lactate dehydrogenase (LDH) record yielded 170 patients, 11 deaths, and AUCs of 0.964 and 0.935, respectively. The combined model predicted 17.8% mortality against 6.5% observed in that subset. **Conclusions:** Flexible effects improved some internal metrics but did not establish an adequate replacement model. Residual misspecification, extrapolation and incompatible predictor timing limit transportability. The calculator implements the linear reference model; definitive admission-time external validation remains necessary.

## 1. Introduction

Estimating mortality risk at hospital admission can support clinical assessment and communication, but the prediction must correspond to a clearly defined outcome and time horizon. Several COVID-19 tools, including the ISARIC 4C Mortality Score and ABC_2_-SPH, combine readily available clinical measurements to estimate in-hospital mortality [1,2]. However, systematic reviews and external evaluations have identified substantial variation in model quality, calibration, and performance across settings [3,4,5]. Discrimination in a development cohort is therefore insufficient evidence for clinical use elsewhere.

In-hospital mortality is also a competing-risk outcome. Discharge alive ends the period during which death can occur within the index admission. Although discharged patients leave the risk set when a death-specific hazard is fitted, absolute mortality probability depends on both death and discharge processes [6,7,8]. A model that estimates only the death hazard cannot obtain the corresponding in-hospital death probability by ignoring discharge. COVID-CALC addressed this issue using a Fine–Gray model, illustrating the relevance of competing risks to bedside prognostication [9].

Existing COVID-19 mortality tools differ in prediction timing, treatment of competing events, validation design, and interpretability. Dynamic reassessment of the 4C Mortality Score evaluates risk as a patient’s condition evolves after admission [10], whereas ABC_2_-SPH is an admission score [2]. COVID-CALC uses a Fine–Gray competing-risk model [9], and ALPACA and CZ-COVID-19 are parsimonious regression-based scores developed with separate validation cohorts [11,12]. Table 1 places these representative approaches alongside the present study. Published results are provided for context and are not head-to-head evidence of superiority.

Artificial intelligence and machine learning are increasingly applied to routinely collected healthcare data for mortality prediction, deterioration detection, discharge planning, and clinical risk stratification. Recent examples include causal policy-learning approaches for ICU discharge and leakage-aware interpretable models for disease-risk screening, illustrating both the opportunities and methodological safeguards required for clinical AI. In COVID-19, both ML and conventional statistical models using routine admission or longitudinal EHR data can achieve useful discrimination, but external and temporal validation frequently reveal reduced transportability and calibration drift. These considerations motivate the present emphasis on an interpretable competing-risk framework, explicit calibration, temporal and external assessment, and decision-analytic evaluation (Supplementary Note S1).

Oxygenation presents a related practical challenge. The arterial oxygen partial pressure to inspired oxygen fraction ratio (PaO_2_/FiO_2_, or P/F) is informative but is not measured in every hospitalized patient. Restricting analysis to arterial measurements can preferentially retain patients who underwent blood-gas assessment. When oxygen saturation and contemporaneous oxygen-delivery information are available, an explicitly defined saturation-based approximation may permit broader inclusion. Such derivation is distinct from statistical imputation and must respect the limits of the underlying relationship [13].

This study aimed to develop and internally evaluate a six-predictor cause-specific model for death within 45 days of admission while accounting for discharge alive and retaining patients whose oxygenation could be derived from documented admission measurements. We retained the original linear model as a reference and, in response to diagnostic concerns, assessed nonlinear and time-varying extensions in a post-hoc reanalysis. Frozen models were then evaluated using a Mexican clinical workbook, with explicit restrictions arising from minimum-oxygenation measurements and reconstructed outcomes. A separate historical comparison used 634 supplied scores, without assuming that their model provenance, horizon or linkage to the workbook had been established. A further objective was to document the offline reference-model calculator. Reporting was guided by TRIPOD and TRIPOD+AI [14,15].

## 2. Materials and Methods

### 2.1. Study Design and Participants

This retrospective prognostic study used a Barcelona hospital cohort for model development and internal validation and independent Mexican data for exploratory model evaluation. The Barcelona source population comprised 2285 patients hospitalized with COVID-19 at Bellvitge University Hospital, Spain, between March 2020 and February 2021. The parent study described adults with SARS-CoV-2 infection confirmed by reverse-transcription polymerase chain reaction [16]. The oxygenation-expanded development analysis required age ≥ 18 years, a hospital outcome with a non-negative elapsed time from admission, and complete values for the six predictors after the oxygenation derivation described below. All eligible patients were considered; no additional sampling or prospective sample-size calculation was undertaken.

The Barcelona cohort has previously been used to describe hospital trajectories in a multistate analysis [16]. The present study addresses a different objective: individualized prediction of 45-day in-hospital mortality from six admission predictors, with bootstrap internal validation and a corresponding calculator. Intensive-care admission was not a competing endpoint because patients remained at risk of death or discharge during the same hospitalization.

Two Mexican sources were distinguished. The clinical workbook from the Monterrey component of the Spanish Coalition to Unlock Research on Host Genetics on COVID-19 (SCOURGE) contained 227 identified records, of which 202 were hospitalized. It supported a patient-level exploratory evaluation subject to predictor and endpoint checks. A separate file contained 634 supplied probability scores and binary hospital outcomes, including 45 deaths; it lacked the individual predictors and linkage needed to rescore those participants with the updated models. The parent Latin-American SCOURGE study recruited between March 2020 and July 2021 [17]. No Mexican outcome was used to estimate, tune or recalibrate the Barcelona models evaluated here. Table 2 distinguishes the sources and their analytical roles.

### 2.2. Ethical Considerations

The Barcelona study was approved by the Bellvitge University Hospital research ethics committee (PR128/20), with a waiver of individual informed consent for retrospective analysis [16]. Recruitment in Monterrey was approved by the Ethics and Research Committee of the School of Medicine, Tecnológico de Monterrey (P000396-GenoCOVID-CEIC-CR002). International collaboration was authorized by Mexico’s Federal Commission for the Protection against Sanitary Risks (COFEPRIS; 203301410A0265/2020). The overarching SCOURGE study was approved by the Galician Ethical Committee (2020/197). Participating SCOURGE centers obtained informed consent for sample and clinical-data collection [17]. Both studies were conducted in accordance with the Declaration of Helsinki.

### 2.3. Outcomes and Follow-Up

Time zero was hospital admission. Let T_i_ denote elapsed time from admission to the first terminal hospital outcome, J_i_ its type (1, in-hospital death; 2, discharge alive), and X_i_ the six-predictor vector for participant i. We retained the manuscript’s prediction horizon of τ = 45 days. The primary binary outcome was death during the index hospitalization by that horizon:

(1) $$\tau =45,\;\;Y_{i}\left(\tau \right)=I\;\left(T_{i}\leq \tau ,\;J_{i}=1\right)$$

Death after discharge was outside this estimand. Final hospital outcome and elapsed time were available for every Barcelona participant included in the analysis. A terminal outcome after day 45 therefore indicated that the participant was alive and still hospitalized at the prediction horizon; it was not treated as an early discharge. Patients discharged alive before day 45 were non-cases for index-admission death.

Both cause-specific models were estimated over the full recorded hospitalization so that all observed terminal events contributed to coefficient and baseline-hazard estimation. Absolute risks were then evaluated at day 45. A sensitivity analysis administratively restricted model estimation to day 45.

### 2.4. Predictors, Oxygenation Derivation, and Missing Data

The six clinically selected Barcelona predictors were recorded sex, age, arterial oxygenation expressed as the PaO_2_/FiO_2_ ratio (P/F), absolute lymphocyte count, Charlson Comorbidity Index (CCI), and lactate dehydrogenase (LDH). Barcelona measurements at admission or within the first 24 h were used; later or worst inpatient measurements were not substituted. Exact within-day ordering between a measurement and an early outcome was unavailable. The clinical rationale is consistent with previous COVID-19 prognostic studies [18,19,20]. The different measurement definitions in the Mexican workbook are specified in Section 2.7.

Sex was coded male = 0 and female = 1. Age was measured in years, P/F in mmHg, lymphocytes in ×10^6^/L (numerically equivalent to cells/µL), CCI in recorded points, and LDH in U/L. All six predictors were retained in both causes without univariable screening or categorization. Age, P/F, lymphocytes, CCI and LDH entered linearly in the reference specification; quadratic terms and time interactions were evaluated in the extensions described below. Age was entered separately from CCI, without adding age points to the recorded CCI.

Oxygenation was determined before predictor completeness was assessed. A recorded arterial P/F value was preferred. When it was absent, P/F could be calculated from paired PaO_2_ and FiO_2_. If arterial information remained unavailable, P/F was approximated from contemporaneous peripheral oxygen saturation (SpO_2_) and FiO_2_ using the inverse Rice relationship [13]:

(2) $$P/F=\frac{{PaO}_{2}}{{FiO}_{2}},\;\;S/F=\frac{{SpO}_{2}}{{FiO}_{2}},\;\;\hat{P/F}=\frac{S/F-64}{0.84}$$

In Equation (2), SpO_2_ is expressed in percentage units and FiO_2_ as a fraction. Saturation-based derivation required SpO_2_ > 0% and ≤97%, a contemporaneous FiO_2_, and a positive derived P/F. FiO_2_ came from a documented concentration or a prespecified mapping of the recorded oxygen-delivery device and flow. Room air was assigned 0.21 only when explicitly recorded.

The Barcelona oxygenation procedure was a measurement derivation, not multiple imputation: it used no outcome, population-average predictor value or later clinical observation. Patients with any required predictor still unavailable after derivation were not included in model fitting. Their available characteristics and outcomes were compared descriptively with those of included patients. No missing-value imputation or outcome-informed predictor replacement was performed. The reference specification and the subsequent diagnostic extensions used the same 1622-patient matrix. However, multiple imputations were only used for sensitivity analysis to demonstrate that the exclusion of patients without complete information does not create selection bias.

### 2.5. Sensitivity Analysis

Because 655 of 2277 eligible Barcelona participants (28.8%) had at least one unresolved model predictor after application of the prespecified oxygenation-reconstruction procedure, a post hoc multiple-imputation sensitivity analysis was performed. Thirty multiply imputed datasets were generated using chained equations with predictive mean matching. The imputation model included all six prognostic-model variables together with auxiliary admission clinical and laboratory variables, admission calendar time, observed follow-up time, event indicators for death and discharge alive, and cause-specific cumulative-hazard information. Outcome information was used only to improve the imputation procedure and was not added as an additional predictor to the prognostic model. The same six-predictor cause-specific Cox specification was refitted in each completed dataset, regression estimates and their uncertainty were combined across imputations, and predictive performance was evaluated from the multiply imputed analyses. As an additional check, the original 1622-patient model was held fixed and applied to imputed predictor values for the 655 participants who had been excluded from the primary analysis.

To assess the potential influence of the SpO_2_/FiO_2_-based Rice approximation, the 1622-patient cohort was divided into 1034 participants with directly measured arterial P/F and 588 participants with P/F derived from contemporaneous SpO_2_ and FiO_2_ using the inverse Rice relationship, restricted to SpO_2_ ≤ 97%. Baseline case mix was compared using standardized mean differences, Mann–Whitney tests for continuous variables, and a chi-square test for recorded sex. Forty-five-day mortality was compared using Fisher’s exact test and a risk ratio with 95% confidence interval. The unchanged CSC45 model was then evaluated separately in each oxygenation subgroup using AUC, average precision, Brier and scaled Brier scores, observed-to-expected ratio, calibration-in-the-large, and calibration slope; subgroup 95% confidence intervals were obtained from 5000 outcome-stratified bootstrap resamples. Between-route AUC heterogeneity was assessed with an outcome-stratified permutation test, and calibration heterogeneity was tested in a logistic calibration model containing logit(predicted risk), oxygenation route, and their interaction. To evaluate whether the prognostic association of P/F differed according to measurement route, cause-specific Cox models included a route indicator and a P/F-by-route interaction and were compared with models without the interaction using likelihood-ratio tests. Finally, the six-predictor cause-specific Cox model was refitted using only the 1034 directly measured-P/F participants and its predictions and coefficients were compared with those from the oxygenation-expanded model.

As a competing-risk sensitivity analysis, we additionally fitted a Fine–Gray proportional subdistribution hazards model for in-hospital death in the same 1622 Barcelona patients, with discharge alive treated as the competing event and the same six predictors retained without further selection or categorization. Whereas the primary cause-specific Cox models estimate instantaneous death and discharge hazards among patients still hospitalized and combine both hazards to obtain the 45-day cumulative incidence of death, the Fine–Gray model directly targets the subdistribution hazard associated with cumulative incidence. Fine–Gray models were fitted over the complete recorded hospitalization, with an additional analysis administratively restricted to 45 days. Predictions from the Fine–Gray and cause-specific approaches were compared using 45-day AUC, average precision, Brier score, mean predicted risk, observed-to-expected ratio, calibration slope, and participant-level agreement, with paired bootstrap resampling used to quantify uncertainty in differences in discrimination and prediction error.

### 2.6. Cause-Specific Models and Absolute Risk

Separate Cox proportional-hazards models were initially fitted for death (k = 1) and discharge alive (k = 2) [21]. These linear models are retained as a reference specification, with their assumption departures reported explicitly. Each contained X = (sex, age, P/F, lymphocytes, CCI, LDH)ᵀ. For estimation of cause k, the alternative terminal event removed a participant from the corresponding risk set at its observed time. For this reference specification, the cause-specific hazard and cumulative hazard were

(3) $$h_{k}\left(t|X\right)=h_{0k}\left(t\right)exp\;\left(\beta_{k}^{T}X\right),\;\;k\in \{1,2\}$$

(4) $$H_{k}\left(t|X\right)=\int_{0}^{t}h_{k}\left(u|X\right)\;du=H_{0k}\left(t\right)exp\;\left(\beta_{k}^{T}X\right)$$

where h_0k_(t) and H_0k_(t) are the cause-specific baseline hazard and cumulative baseline hazard, and β_k_ is the coefficient vector. Tied event times were handled by the Efron approximation [22]. At event time u, let R(u) = {i: T_i_ ≥ u} denote the risk set, D_k_(u) = {i: T_i_ = u, J_i_ = k} the cause-specific event set, d_k_(u) its size, and η_ki_ = β_k_ᵀX_i_. The risk-set and tied-event sums were

(5) $$\begin{array}{ll} d_{k}\left(u\right) & =\left|D_{k}\left(u\right)\right|,\\ R_{k}^{\left(0\right)}\left(u;\beta_{k}\right) & =\sum_{j\in R\left(u\right)}e^{\eta_{kj}},\;\;E_{k}^{\left(0\right)}\left(u;\beta_{k}\right)=\sum_{i\in D_{k}\left(u\right)}e^{\eta_{ki}} \end{array}$$

with U_k_ denoting the distinct event times for cause k. The Efron partial log likelihood maximized for each cause was

(6) $$l_{k}\left(\beta_{k}\right)=\sum_{u\in U_{k}}\left\{\sum_{i\in D_{k}\left(u\right)}\eta_{ki},-,\sum_{l=0}^{d_{k}\left(u\right)-1}log,\;,\left[R_{k}^{\left(0\right)}\left(u;\beta_{k}\right)-\frac{l}{d_{k}\left(u\right)}E_{k}^{\left(0\right)}\left(u;\beta_{k}\right)\right]\right\}$$

The model-based covariance matrix was the inverse observed information at the maximum. For predictor q and reporting increment Δxq, adjusted cause-specific hazard ratios, Wald confidence intervals, test statistics, and two-sided *p* values were calculated as

(7) $$\begin{array}{ll} {\hat{HR}}_{kq}\left(\Delta x_{q}\right) & =exp\;\left(\Delta x_{q}{\hat{\beta }}_{kq}\right),\\ {CI}_{95\%,kq}\left(\Delta x_{q}\right) & =exp\;\left\{\Delta x_{q}{\hat{\beta }}_{kq}\pm z_{0.975}\left|\Delta x_{q}\right|\;SE\left({\hat{\beta }}_{kq}\right)\right\} \end{array}$$

(8) $$z_{kq}^{W}=\frac{{\hat{\beta }}_{kq}}{SE\left({\hat{\beta }}_{kq}\right)},\;\;p_{kq}^{W}=2\left[1-\Phi \;\left(\left|z_{kq}^{W}\right|\right)\right]$$

where Φ is the standard normal distribution function and z_0.975_ = 1.96. Reporting increments were 10 years for age, 100 mmHg for P/F, 500 × 10^6^/L for lymphocytes, one CCI point and 100 U/L for LDH; sex compared female with male. Scaling changed only the reported contrast in the reference model. Its hazard ratios are working constant-effect summaries of conditional associations, not risk ratios, causal effects or evidence of time-invariant biological effects. In the nonlinear, time-varying extension, a contrast depends on the predictor values and follow-up time.

The two fitted hazards were combined to estimate absolute outcome probabilities [23,24]. The event-free probability S(t | X), cumulative incidence F_k_(t | X), and probability identity were

(9) $$S\left(t|X\right)=exp\;\left\{-H_{1}\left(t|X\right)-H_{2}\left(t|X\right)\right\}$$

(10) $$F_{k}\left(t|X\right)=\int_{0}^{t}S\left(u^{-}|X\right)\;dH_{k}\left(u|X\right),\;\;k\in \{1,2\}$$

(11) $$F_{1}\left(t|X\right)+F_{2}\left(t|X\right)+S\left(t|X\right)=1$$

For numerical prediction from the linear reference model, c_k_ is the stored centering vector and uᵣ denotes an ordered event-grid point through day 45. The centered linear predictor, cause-specific hazard increments and their sum were

(12) $$\begin{array}{ll} {\hat{\eta }}_{k}\left(X\right) & ={\hat{\beta }}_{k}^{T}\left(X-c_{k}\right),\\ a_{kr}\left(X\right) & =exp\;\left\{{\hat{\eta }}_{k}\left(X\right)\right\}\Delta {\hat{H}}_{0k}^{\left(c_{k}\right)}\left(u_{r}\right),\;\;A_{r}\left(X\right)=a_{1r}\left(X\right)+a_{2r}\left(X\right) \end{array}$$

Starting immediately before the first grid point with ${\hat{F}}_{1,0}$ = ${\hat{F}}_{2,0}$ = 0 and Ŝ_0_ = 1, the exponential within-interval competing-hazard update was

(13) $$\begin{array}{rr} {\hat{F}}_{k,r} & ={\hat{F}}_{k,r-1}+{\hat{S}}_{r-1}\left[1-e^{-A_{r}}\right]\frac{a_{kr}}{A_{r}},\;\;k\in \{1,2\},\\ {\hat{S}}_{r} & ={\hat{S}}_{r-1}e^{-A_{r}} \end{array}$$

When Aᵣ = 0, all three probabilities remain unchanged. The update integrates the combined fitted hazard over each recorded interval and assigns the interval event probability according to the two cause-specific hazard contributions. It preserves non-negativity and ${\hat{F}}_{1,r}$ + ${\hat{F}}_{2,r}$ + Ŝᵣ = 1 without clipping or renormalization. Mortality risk was ${\hat{F}}_{1}$(45 | X); it was not calculated from the death hazard alone.

Post-hoc extensions retained the six predictors and separately added five quadratic continuous-predictor terms, six interactions with log(1 + t), or both. For the combined quadratic plus time-varying (QTV) model, the linear predictor was

(14) $$\eta_{k}(t,\;X)=\beta_{k}^{T}W+\sum_{j\;\in \;C}\frac{\alpha_{kj}W_{j}^{2}}{q_{j}}+log(1+t)\;\gamma_{k}^{T}W$$

Here W_j_ = (X_j_ − c_j_)/s_j_, with Barcelona training means c_j_ for continuous predictors and csex = 0. Scaling increments s_j_ were 1, 10 years, 100 mmHg, 500 cells/µL, one CCI point and 100 U/L. C comprises the five continuous predictors, and q_j_ is the training-sample standard deviation of W_j_^2^, used for numerical conditioning. The candidate has 17 coefficients per cause: six main effects, five quadratic terms and six time interactions. Quadratic coefficients are constant over time in this specification; setting α or γ to zero gives the simpler extensions.

These are time-varying coefficients applied to fixed baseline measurements. At each event time u, log(1 + u) was evaluated for every member of that risk set; an individual’s future terminal time was never entered as a baseline predictor. Both causes were refitted over full hospitalization follow-up. The frozen time-specific linear predictor and its corresponding baseline increment replaced the reference quantities in Equation (12), with the same competing-hazard update in Equation (13).

### 2.7. Performance, Internal Validation, and Model Diagnostics

For Barcelona, y_i_ = Y_i_(45) and p_i_ = ${\hat{F}}_{1}$(45 | X_i_). The numbers of cases and non-cases are denoted n_1_ and n_0_. Because day-45 status was known from the terminal outcome and follow-up time for every included participant, ordinary binary performance measures were applicable at this horizon. A cohort with unknown status at day 45 would require censoring-aware measures [25].

Discrimination was summarized by the area under the receiver operating characteristic curve (AUC), with half credit for tied predictions:

(15) $$\hat{AUC}=\frac{1}{n_{1}n_{0}}\sum_{i:y_{i}=1}\sum_{j:y_{j}=0}\left[I\left(p_{i}>p_{j}\right)+\frac{1}{2}I\left(p_{i}=p_{j}\right)\right]$$

Average precision (AP) was the recall-increment-weighted mean of precision across the L distinct prediction thresholds, rather than a trapezoidal precision–recall area:

(16) $$\hat{AP}=\sum_{l=1}^{L}\left(R_{l}-R_{l-1}\right)P_{l},\;\;R_{0}=0$$

Overall prediction error was evaluated with the Brier score (BS) and a scaled Brier score relative to constant prediction of the observed event proportion:

(17) $$\begin{array}{rr} \hat{BS} & =\frac{1}{n}\sum_{i=1}^{n}{\left(y_{i}-p_{i}\right)}^{2},\;\;\hat{\pi }=\frac{1}{n}\sum_{i=1}^{n}y_{i},\\ {\hat{BS}}_{scaled} & =1-\frac{\hat{BS}}{\hat{\pi }\left(1-\hat{\pi }\right)} \end{array}$$

Calibration was summarized by the mean predicted probability, observed-to-expected ratio, calibration-in-the-large (CITL), and a logistic calibration model with a freely estimated intercept and slope [26]:

(18) $$\bar{p}=\frac{1}{n}\sum_{i=1}^{n}p_{i},\;\;O/E=\frac{\sum_{i=1}^{n}y_{i}}{\sum_{i=1}^{n}p_{i}}$$

(19) $$\begin{array}{ll} logitPr\left(Y_{i}=1|z_{i}\right) & =\alpha_{CITL}+offset\left(z_{i}\right),\\ logitPr\left(Y_{i}=1|z_{i}\right) & =\alpha_{cal}+\gamma_{cal}z_{i},\;\;z_{i}=logit\left(p_{i}\right) \end{array}$$

Apparent performance was evaluated in the 1622-patient development cohort. Conditional 95% confidence intervals used B = 2000 outcome-stratified bootstrap resamples of the fixed predictions, preserving 325 deaths and 1297 non-deaths. For any statistic θ, the percentile interval was

(20) $${CI}_{95\%}\left(\hat{\theta }\right)=\left[Q_{0.025}\;\left({\hat{\theta }}^{*\left(1\right)},\ldots ,{\hat{\theta }}^{*\left(B\right)}\right),\;Q_{0.975}\;\left({\hat{\theta }}^{*\left(1\right)},\ldots ,{\hat{\theta }}^{*\left(B\right)}\right)\right]$$

The pointwise 95% confidence band applies Equation (19) to sensitivity at each fixed false-positive-rate grid point. These intervals condition on the fitted model and observed event proportion; they do not include refitting or model-selection uncertainty. Calibration plots used ten approximately equal-count risk groups with Wilson 95% intervals for the observed proportions [27].

Internal validation of the linear reference model used B = 1000 unstratified patient-level bootstrap resamples [28]. Both cause-specific models were refitted in every resample and evaluated in the bootstrap sample and the original cohort. If ${\hat{\theta }}_{boot,b}$ and ${\hat{\theta }}_{orig,b}$ are the corresponding estimates from bootstrap model b, optimism and the corrected estimate were

(21) $$\begin{array}{ll} O_{b} & ={\hat{\theta }}_{boot,b}-{\hat{\theta }}_{orig,b},\\ {\hat{\theta }}_{corrected} & ={\hat{\theta }}_{apparent}-\frac{1}{B}\sum_{b=1}^{B}O_{b} \end{array}$$

The same optimism sign convention was used for every metric, including the Brier score. The 1000-bootstrap analysis applies only to the linear reference model. PH was assessed by Efron-adjusted joint score tests for interactions between all six predictors and log(1 + time), accounting for fitted main effects [29]. Linearity was examined by joint likelihood-ratio tests adding five quadratic continuous-predictor terms. Scaled Schoenfeld residuals plus fitted coefficients were smoothed with natural cubic splines using four degrees of freedom, with approximate ±2 standard-error bands. Main plots display days 0–45, with smoothers estimated over full follow-up. PH tests were repeated with estimation restricted to day 45, excluding day-zero outcomes, and using identity and rank time transformations. Other reference-model sensitivities used measured arterial P/F only and excluded extreme lymphocyte values.

The four fixed specifications were compared using three repetitions of five-fold cross-validation, with common folds stratified by terminal cause. Both hazards, centering constants and quadratic scaling factors were estimated within each training fold; only held-out patients contributed to each validation prediction. We report means of the three complete out-of-fold performance estimates. This analysis does not reproduce the entire post-hoc choice of model class. Paired conditional bootstrap contrasts in Supplement S2 keep fitted out-of-fold predictions and partitions fixed and do not constitute equivalence tests.

Remaining restrictions of the QTV model were explored by conditional score tests for six additional predictor-by-log^2^(1 + t) terms, five quadratic-by-log(1 + t) terms and five cubic predictor terms, accounting for all 17 fitted coefficients. These tests examine specified residual departures; non-significance does not prove adequacy. The extensions were assessed as sensitivity analyses, and the original linear model was retained as a reference rather than declared assumption-compliant.

### 2.8. Exploratory Evaluation Using Mexican Data

The historical supplied-score analysis used recorded in-hospital mortality Z_i_, a supplied risk p_i_^B^ labelled as the Barcelona score, and a supplied COVID-CALC score p_i_^C^. Its prediction horizon, model provenance and patient linkage could not be independently established from the available score file. The paired AUC contrast was defined as

(22) $$\Delta_{AUC}=\hat{AUC}\left(p^{B},Z\right)-\hat{AUC}\left(p^{C},Z\right)$$

Historical AUC, AP, Brier score and calibration were summarized in the same 634 score records. DeLong’s paired test assessed the AUC contrast [30]; percentile intervals for performance and paired AP and Brier differences used 5000 outcome-stratified paired resamples. These are descriptive supplied-score comparisons against recorded hospital mortality. They do not establish external validation of the expanded reference model or any diagnostic extension, and their calibration and prediction-error comparisons do not use a verified common 45-day endpoint.

For the clinical workbook, identified hospitalized records from the primary sheet were checked against the secondary sheet by exact patient identifier, without appending duplicate records. Sex was mapped to female = 1; age, lymphocytes, recorded CCI and LDH were extracted from the corresponding fields. The dictionary identified lactato as LDH in U/L. Oxygenation was approximated as (minimum S/F − 64)/0.84. Recorded minimum saturation had to lie in (0,97], derived P/F had to be positive, and minimum S/F could not be below recorded minimum saturation. These restrictions do not establish paired measurements or admission timing; lymphocyte-unit and CCI compatibility also remain assumptions.

The workbook target was index-admission death by day 45. Non-events were supported by documented live discharge or recorded survival at 90 days after diagnosis extending through the admission-based horizon. For death exits, available plausible stored durations and admission-to-exit intervals had to agree on horizon classification. A valid day/month reversal was considered only as a diagnostic candidate when the recorded exit preceded admission, without changing the source. Invalid admission years and horizon-discordant death records were excluded. Reconstruction assumes accurate survival ascertainment and that the recorded death exit belongs to the index admission. Descriptive length of stay (LOS) used a separately identified set after two evident duration/date errors were excluded; it was not used to retrospectively claim prospective selection of the horizon.

The primary source-eligible scoring set applied these endpoint checks and excluded missing predictors, unsupported oxygenation and conflicting LDH across sheets. It retained a recorded LDH of 24,181 U/L, with a separate sensitivity excluding that record. This differs from the previous original-model evaluation, where that record produced an invalid product-limit prediction. The expanded linear and QTV models were both frozen from the same 1622 Barcelona patients and used exponential competing-risk integration without Mexican refitting, tuning or recalibration. Both differ from the older 1035-patient saved CSC. No linked, horizon-matched COVID-CALC probabilities were available for the workbook cohort.

All retained day-45 outcomes were classifiable under the stated reconstruction, so discharged survivors were non-cases and binary discrimination, Brier, O/E and calibration measures were used without censoring weights. Uncertainty for the 171- and 170-person analyses used 5000 ordinary paired patient bootstrap resamples, with frozen Barcelona models and explicit counts of finite calibration estimates. Calibration regressions assessed the predictions and did not update them. Quartile calibration points used Wilson intervals. Other fixed sensitivities considered both interpretations of the ambiguous 43/48-day death, either LDH sheet for conflicting records, an alternative S/F unit convention, hemograms dated on admission day or the next calendar day, and 28- and 30-day horizons. These exploratory scenarios were not chosen according to their performance.

### 2.9. Temporal Internal Validation

Temporal performance was evaluated according to prespecified pandemic-wave periods defined independently of model predictions and outcomes. For the primary analysis, calendar periods followed the Catalonia epidemiological-wave definition: Wave 1 from 1 March through 30 September 2020, Wave 2 from 1 October through 9 December 2020, and Wave 3 from 10 December 2020 through 14 March 2021. Because Barcelona recruitment in the present dataset ended on 13 February 2021, the third-wave validation period was truncated at that date. Four patients admitted before 1 March 2020 were excluded only from the wave-stratified temporal analysis. Two complementary temporal assessments were performed.

First, the frozen six-predictor CSC45 model fitted in the complete 1622-patient development cohort was evaluated separately within each pandemic wave. For each wave we calculated the observed 45-day mortality proportion, mean predicted 45-day mortality, AUC, average precision, Brier score, observed-to-expected ratio, calibration-in-the-large, and calibration slope. Pointwise 95% confidence intervals were obtained from 2000 outcome-stratified bootstrap resamples of the fixed wave-specific predictions.

Between-wave discrimination heterogeneity was assessed using an outcome-stratified permutation test of the maximum minus minimum AUC across the three waves. Calibration-intercept heterogeneity was assessed by comparing logistic calibration models with and without wave-specific intercept terms using a likelihood-ratio test. Calibration-slope heterogeneity was assessed by adding wave-by-logit-prediction interaction terms.

Second, a chronological temporal hold-out analysis was performed to more closely mimic prospective transport over calendar time. The same prespecified six-predictor cause-specific Cox specification was fitted using only Wave-1 patients and applied without recalibration to Wave 2 and, separately, to Wave 3. A further model was fitted using Waves 1 and 2 combined and applied without updating to Wave 3. Cause-specific models for death and discharge alive were refitted only within the designated training period; 45-day cumulative mortality probabilities in the later validation period were calculated by combining both cause-specific hazards using the same competing-risk integration procedure as in the primary model.

As a sensitivity analysis, performance was also examined within narrower published Barcelona metropolitan south/Bellvitge wave windows: 1 March–15 April 2020; 1 October–30 November 2020; and 1 January–28 February 2021.

### 2.10. Decision Curve Analysis

Clinical utility was examined using decision curve analysis at the 45-day prediction horizon. The event was in-hospital death by day 45; participants discharged alive before day 45 or remaining alive without the event at day 45 were classified as non-events for this fixed-horizon analysis. The predicted probability was the 45-day cumulative incidence of death obtained from the two cause-specific Cox processes, thereby accounting for discharge alive as a competing event in generation of the individual risk predictions.

For a threshold probability ($p_{t}$), net benefit was calculated as:

(23) $$NB\left(p_{t}\right)=\frac{TP}{N}-\frac{FP}{N}\times \left(\frac{p_{t}}{1-p_{t}}\right)$$

where TP and FP denote the numbers of true- and false-positive classifications at that threshold. The model was compared with the standard treat-all and treat-none reference strategies across threshold probabilities from 0.05 to 0.50. The displayed threshold range is exploratory and does not constitute a recommended clinical treatment or triage range.

To reduce same-sample optimism, decision curves were additionally calculated from three repetitions of 5-fold cross-validation. Within each repetition, the same prespecified six-predictor cause-specific Cox models for death and discharge alive were refitted in the training folds and 45-day mortality probabilities were generated only for held-out participants. Net benefit was calculated separately for each repetition and averaged across repetitions. Pointwise 95% intervals were obtained from 2000 patient-level bootstrap resamples conditional on the stored out-of-fold predictions and fold assignments; these intervals therefore quantify sampling uncertainty in the decision curves but do not constitute a nested-bootstrap estimate of the entire model-development process.

### 2.11. Software and Calculator

Analyses used Python (v3.13.5) with NumPy (v2.3.5), SciPy (v1.17.0), scikit-learn (v1.8.0), and statsmodels (v0.14.6); exact runtime versions are retained with the analysis files. Reference-model Efron estimates were cross-checked against statsmodels. The quadratic plus time-varying coefficients were independently reproduced using a separate Efron implementation on event-split risk intervals, following the time-dependent coefficient formulation [31]. Frozen-parameter scoring was checked against all 1622 Barcelona predictions. Mexican discrimination and Brier scores were checked against scikit-learn and calibration regressions against statsmodels. The offline JavaScript (https://developer.mozilla.org/en-US/docs/Web/JavaScript; accessed 17 September 2026) calculator continues to implement the expanded linear reference model, using its coefficients, centering constants and baseline hazards. Its original 20-profile numerical and input checks are distinct from validation of the flexible extension.

## 3. Results

### 3.1. Study Population and Oxygenation Availability

Of the 2285 Barcelona patients assessed, one was younger than 18 years and seven adults had a terminal hospital date preceding admission, leaving 2277 eligible adults. Following oxygenation derivation, 1622 patients (71.2%) had complete information for all six predictors. Of these, 1034 (63.7%) had measured arterial P/F and 588 (36.3%) had saturation-derived P/F. No additional patient with an absent P/F had sufficient paired arterial measurements to contribute through the PaO_2_/FiO_2_ calculation alone.

Saturation-based derivation provided an oxygenation value for 684 eligible patients; 96 still lacked another required predictor. Among all 2277 eligible adults, P/F remained unavailable in 440 (19.3%), LDH in 269 (11.8%), and lymphocyte count in 18 (0.8%), with overlap. Thus, 655 patients (28.8%) were excluded for remaining predictor incompleteness. The absence of a directly entered FiO_2_ or arterial P/F did not by itself determine exclusion.

The analysis population included 597 women (36.8%); median age was 68.8 years (interquartile range [IQR], 56.8–77.7). Median P/F was 338.1 mmHg (IQR, 271.4–462.2), lymphocyte count 890 × 10^6^/L (IQR, 633–1223), and LDH 325 U/L (IQR, 264–421). By day 45, 325 patients (20.0%) had died, 1204 (74.2%) had been discharged alive, and 93 (5.7%) remained hospitalized. Across the full observed hospitalization, there were 346 deaths and 1276 discharges. Three outcomes occurred on the admission day: two deaths and one discharge.

Patients with saturation-derived P/F had lower 45-day mortality than those with measured arterial P/F (11.7% versus 24.8%) and lower median LDH (292.5 versus 348.0 U/L). Patients excluded for residual predictor incompleteness were younger and had lower observed mortality than included patients (13.0% versus 20.0%). Table 3 summarizes these differences; they indicate that predictor availability was associated with clinical case mix.

### 3.2. Cause-Specific Associations

Under the working linear PH reference specification, older age, lower P/F, higher CCI and higher LDH were associated with a greater death-specific hazard. Per 10-year age increment, the adjusted summary hazard ratio was 1.859 (95% CI, 1.678–2.061); per 100-mmHg P/F increment it was 0.672 (0.612–0.738). For discharge, higher P/F had a positive summary association, whereas age, CCI and LDH had negative summary associations. All six predictors were retained (Table 4). Because PH and linearity departures were detected, these estimates summarize the reference fit and should not be interpreted as constant effects throughout follow-up.

### 3.3. Discrimination, Calibration, and Internal Validation

**Figure 1:**
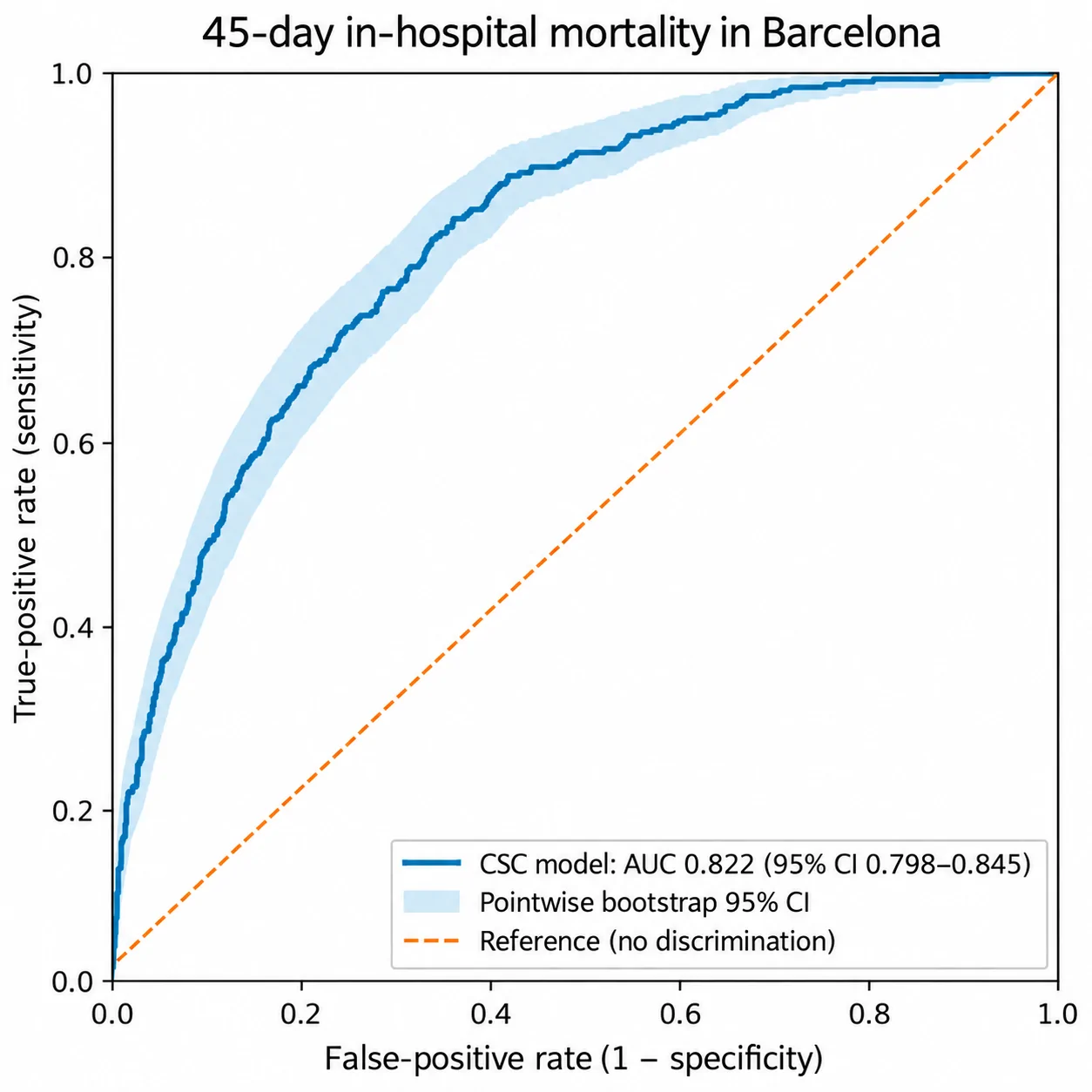
Apparent discrimination of the expanded linear reference model for 45-day in-hospital mortality in 1622 Barcelona patients. The solid curve is the empirical ROC curve; the shaded region is a pointwise 95% band from 2000 outcome-stratified resamples of fixed predictions. AUC was 0.822 (conditional 95% CI, 0.798–0.845). The diagonal indicates chance discrimination.

**Figure 2:**
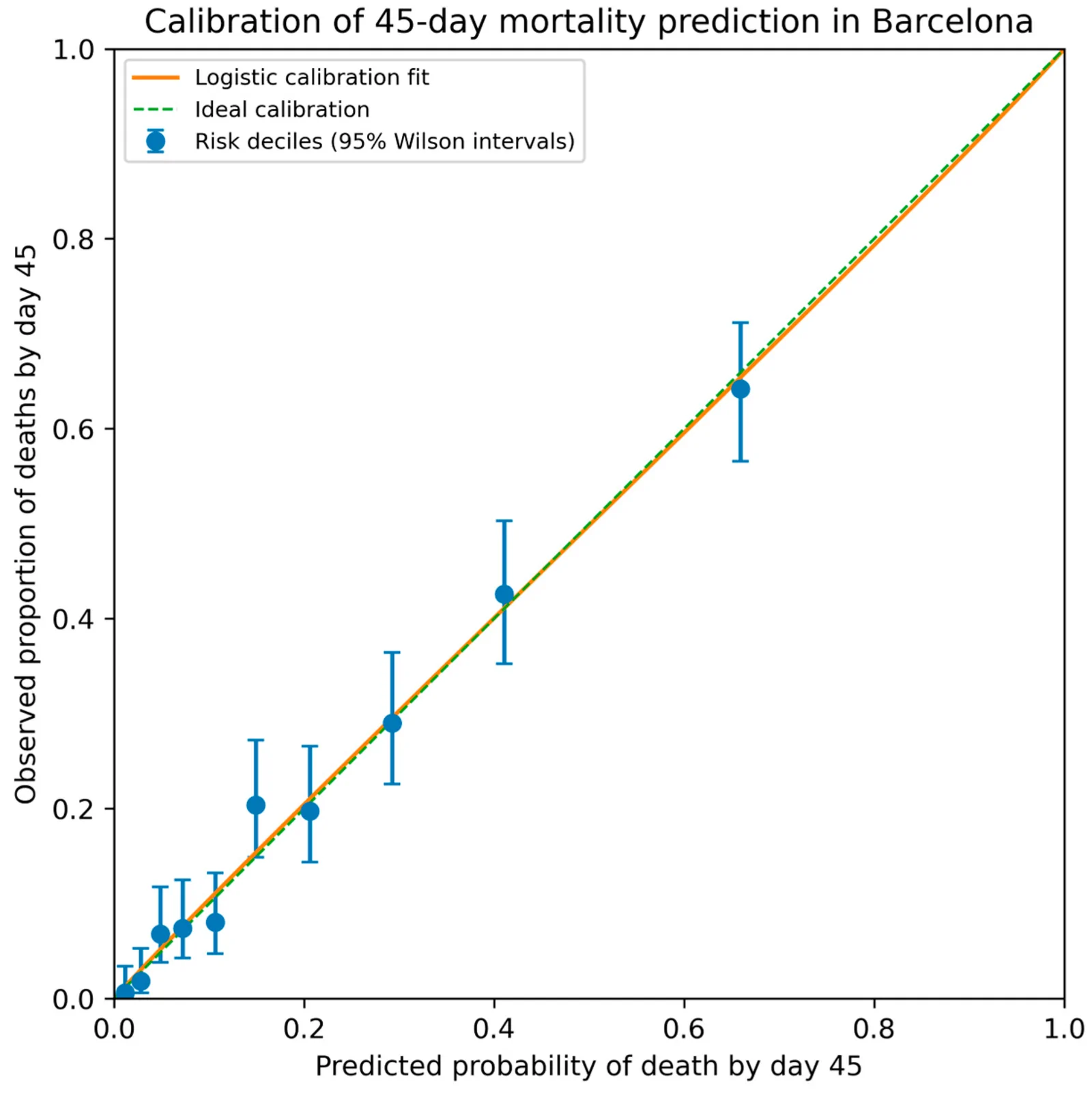
Apparent calibration of the expanded linear reference model in Barcelona. Points are observed mortality and mean predicted risk within deciles, with Wilson 95% intervals. The logistic calibration curve is fitted in the development cohort; this is neither external calibration nor an assessment of the flexible extension.

For the expanded linear reference model, apparent 45-day AUC was 0.822 (95% CI, 0.798–0.845), with the empirical ROC curve and pointwise bootstrap band shown in Figure 1. AP was 0.551 (0.503–0.604), Brier score 0.122 (0.115–0.130) and scaled Brier score 0.236. Mean predicted mortality was 19.8% against 20.0% observed; O/E was 1.011, CITL 0.019 and calibration slope 0.975 (Table 5 and Figure 2).

Both reference hazards were estimated in all 1000 internal-validation resamples. Mean AUC optimism was 0.002, giving corrected AUC 0.820. Corrected AP was 0.542, Brier score 0.123 and calibration slope 0.958 (Table 5). These estimates describe the original fixed linear specification and oxygenation rule; they neither validate the later extensions nor measure transportability. The model comparisons below use separate repeated cross-validation.

### 3.4. Model Diagnostics and Sensitivity Analyses

**Figure 3:**
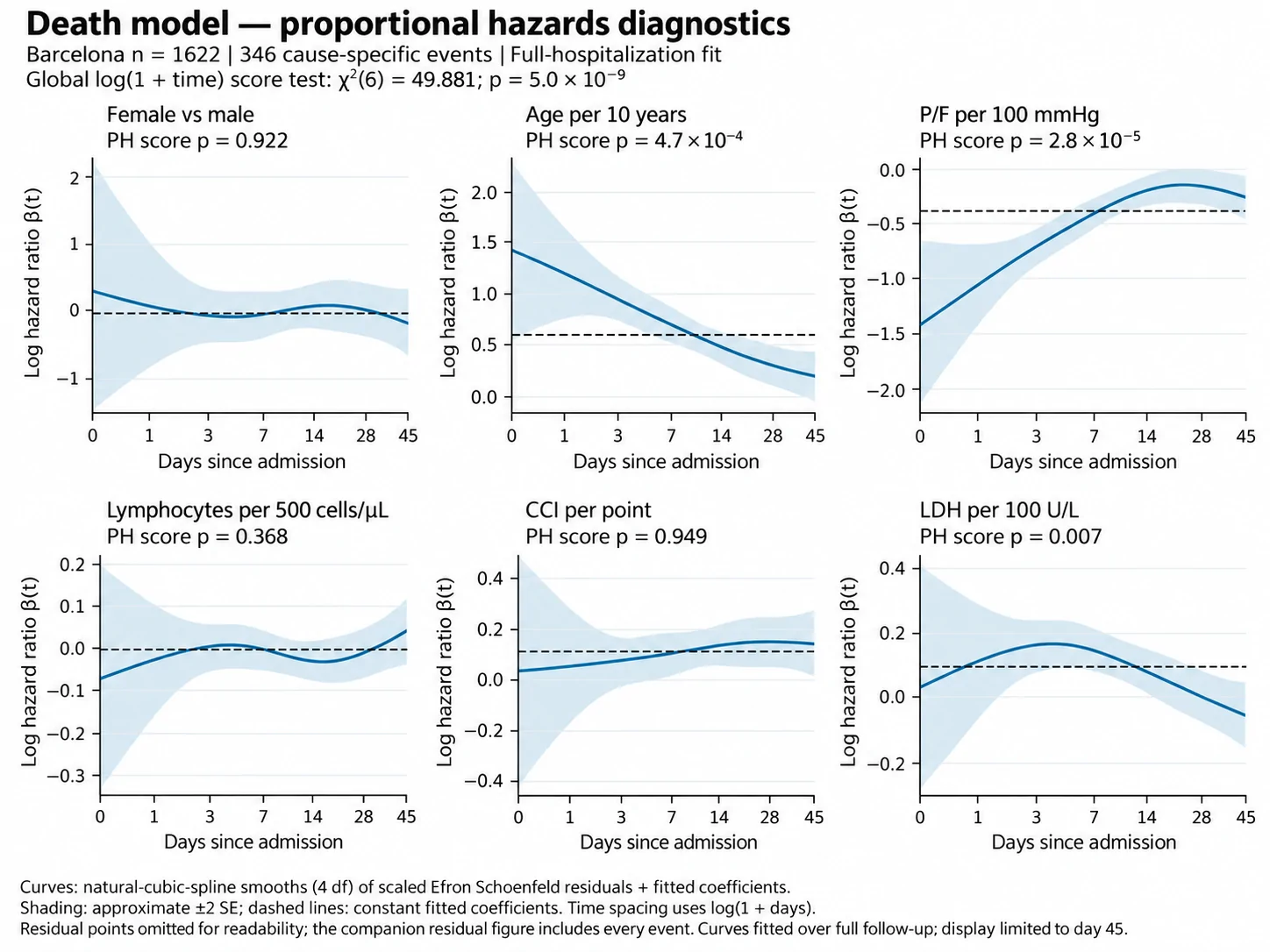
PH diagnostics for death in the Barcelona reference model. Curves are smoothed scaled Schoenfeld residuals plus fitted coefficients; shaded areas are approximate ±2 standard errors and dashed horizontal lines are fitted constant coefficients. The log(1 + days) axis is labelled in days. The display spans 0–45 days, but smoothing uses all 346 deaths over full follow-up. Individual *p* values are unadjusted score tests. Full-follow-up and all-residual plots are in Supplement S2.

**Figure 4:**
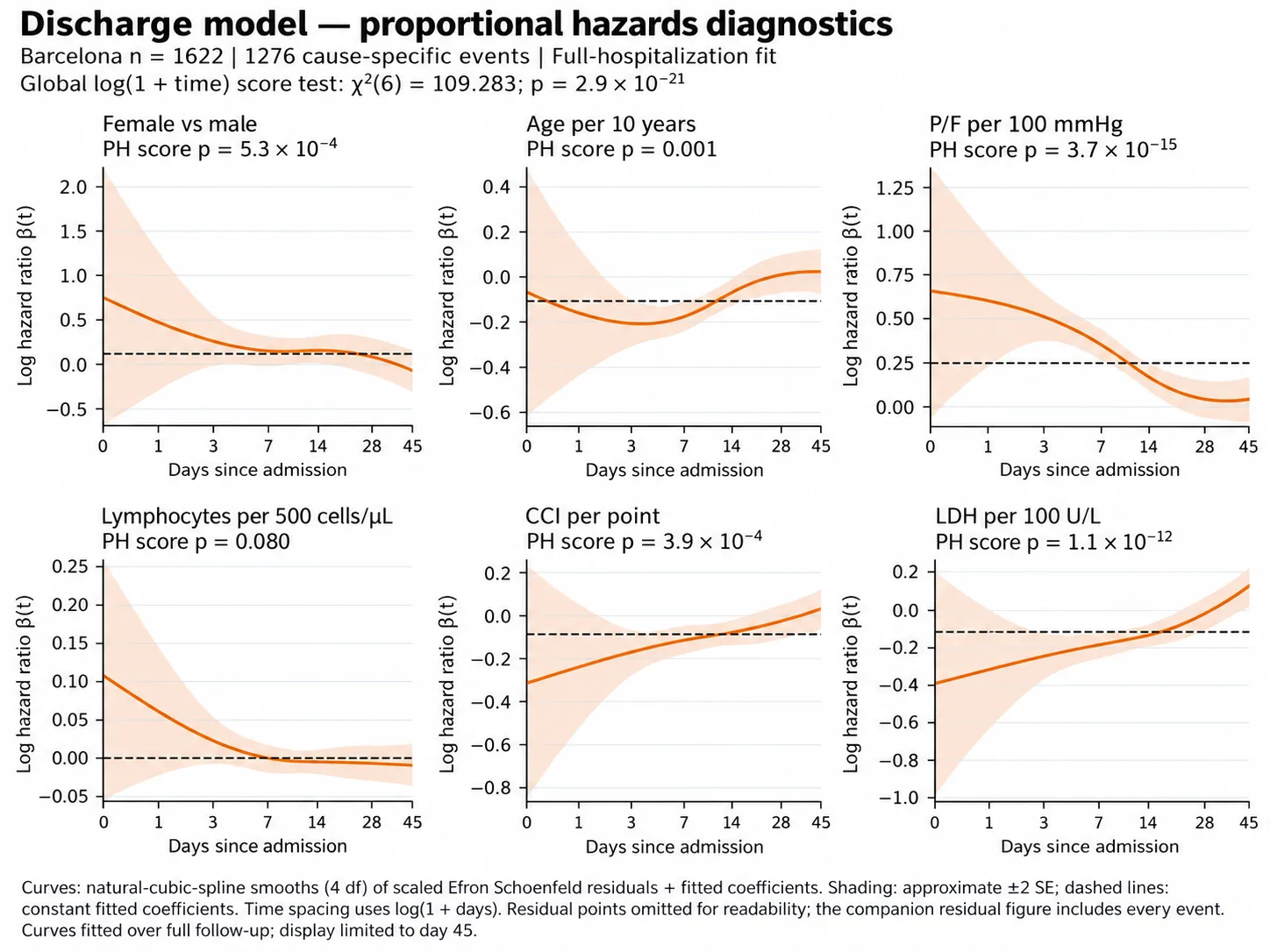
PH diagnostics for discharge alive using the same conventions as Figure 3. Smoothing uses all 1276 discharges; the main view displays days 0–45. Non-horizontal curves indicate possible time-varying effects. Bands are approximate diagnostic intervals, not simultaneous confidence bands. At days 0, 7, 14, 28 and immediately before day 45, the common hospital risk set contained 1622, 1205, 573, 225 and 95 patients, respectively.

The original diagnostic results were reproduced. Global PH score statistics were χ^2^(6) = 49.881 for death (*p* = 4.97 × 10^−9^) and 109.283 for discharge (*p* = 2.88 × 10^−21^). Departures persisted when estimation was restricted to 45 days and after excluding day-zero outcomes (Table 6); identity-time and rank-time tests also rejected PH. Mortality departures were most evident for age (*p* = 0.000474), P/F (*p* = 0.0000284) and LDH (*p* = 0.00711), each remaining significant after Holm adjustment within the six predictors. For discharge, sex, age, P/F, CCI and LDH showed time variation. Figure 3 and Figure 4 display the diagnostic curves. Joint quadratic-term tests were significant for death, χ^2^(5) = 29.303 (*p* = 2.02 × 10^−5^), and discharge, χ^2^(5) = 13.403 (*p* = 0.0199). These results preclude treating either original assumption as satisfied.

Across repeated cross-validation, the quadratic model had AUC 0.8296 and Brier 0.12017, compared with 0.8193 and 0.12343 for the reference. Time variation alone gave AUC 0.8223 and Brier 0.12273. The combined QTV model had AUC 0.8307 and Brier 0.11974, but calibration slope 0.830 versus 0.945 for the reference (Table 7). Its apparent full-data AUC was 0.8344 and Brier 0.11765. In the full-data linear time-varying fit, absolute changes in individual risk relative to the reference had median 0.80 percentage points, 95th percentile 2.92 points and maximum 7.71 points.

Both term sets improved likelihood when added conditionally to the other: for death, χ^2^(6) = 50.641 for time interactions and χ^2^(5) = 32.413 for quadratic effects; for discharge, corresponding statistics were 132.000 and 41.410 (all *p* < 0.001). Nevertheless, conditional score diagnostics beyond the combined model identified residual discharge nonlinearity and time variation of quadratic terms (Table 8). Thus the combined extension relaxes the original restrictions but does not establish a fully adequate functional form.

Reference-model sensitivities showed apparent AUC 0.817 and Brier 0.139 when fitting only the 1034 measured-P/F patients. Reference predictions had AUC 0.818 in measured-P/F patients and 0.792 in saturation-derived patients; these are case-mix-dependent subgroup results, not independent validations. Excluding day-zero outcomes yielded AUC 0.821 and Brier 0.122; restricting estimation to day 45 yielded 0.824 and 0.122; excluding three very high lymphocyte measurements yielded 0.828 and 0.121.

Among the 2277 eligible Barcelona participants, 1622 were included in the primary analysis and 655 (28.8%) had at least one unresolved model predictor. The excluded participants were younger (mean age, 63.5 vs. 67.1 years), had lower observed LDH concentrations (279.6 vs. 359.5 U/L), and had lower 45-day mortality (85/655 [13.0%] vs. 325/1622 [20.0%]). In the multiple-imputation analysis of all 2277 participants, pooled patient-level predictions yielded an AUC of 0.830 (95% CI, 0.809–0.850), Brier score of 0.113 (95% CI, 0.107–0.119), observed-to-expected ratio of 0.994 (95% CI, 0.954–1.030), and calibration slope of 0.962 (95% CI, 0.866–1.062). These estimates were close to the primary analysis values (AUC, 0.822; Brier score, 0.122; observed-to-expected ratio, 1.011; calibration slope, 0.975). Within the original 1622 participants, mortality probabilities from the imputed and primary analyses were highly concordant (Pearson r = 0.997), with a mean absolute difference of approximately 0.01. When the original model was held fixed and applied after imputation to the 655 previously excluded participants, AUC was 0.835 (95% CI, 0.794–0.875), Brier score was 0.093, and the observed-to-expected ratio was 1.029 (95% CI, 0.953–1.111).

The directly measured-P/F subgroup included 1034 participants with 256 deaths by day 45 (24.8%), whereas the Rice-derived subgroup included 588 participants with 69 deaths (11.7%); the derived-to-measured mortality risk ratio was 0.474 (95% CI, 0.371–0.606; *p* < 0.001). The two groups differed markedly in case mix: median P/F was 295.2 [233.3–342.9] versus 465.2 [451.0–473.7] mmHg (absolute SMD, 1.81; *p* < 0.001), and median LDH was 348 [273–452] versus 292.5 [242–360.2] U/L (SMD, 0.46; *p* < 0.001). Despite these differences, discrimination remained similar: AUC was 0.818 (95% CI, 0.789–0.846) in the measured subgroup and 0.792 (95% CI, 0.741–0.840) in the Rice-derived subgroup. The derived-minus-measured AUC difference was −0.025 (bootstrap 95% CI, −0.085 to +0.031; permutation *p* = 0.382). Mean predicted versus observed mortality was 24.6% versus 24.8% in the measured subgroup and 11.4% versus 11.7% in the derived subgroup; observed-to-expected ratios were 1.006 and 1.033, and calibration slopes were 1.023 (95% CI, 0.882–1.184) and 0.859 (95% CI, 0.664–1.103), respectively. There was no evidence of route-specific calibration-intercept heterogeneity (*p* = 0.408) or calibration-slope heterogeneity (*p* = 0.247).

For the death-specific hazard, the adjusted HR per 100-mmHg higher P/F was 0.652 (95% CI, 0.571–0.744) in the measured subgroup and 0.821 (95% CI, 0.670–1.006) in the Rice-derived subgroup. The likelihood-ratio test for the P/F-by-route interaction was borderline (interaction HR ratio, 1.259; 95% Wald CI, 0.994–1.595; *p* = 0.0477), whereas no corresponding interaction was identified for discharge alive (interaction HR ratio, 1.059; 95% CI, 0.888–1.264; *p* = 0.515). Re-fitting the six-predictor model using only the 1034 directly measured-P/F participants yielded almost unchanged overall performance when applied to the full cohort (AUC, 0.820 vs. 0.822; Brier score, 0.12242 vs. 0.12240). Individual 45-day risks from the measured-only and oxygenation-expanded fits correlated at r = 0.993, with a mean absolute difference of 1.67 percentage points; 51 of 1622 participants (3.1%) changed classification at a 20% risk threshold, and all clinically scaled hazard-ratio contrasts changed by less than 9%.

The Fine–Gray sensitivity model included the same 1622 patients as the primary cause-specific analysis. There were 346 deaths over complete recorded hospitalization, of which 325 occurred by day 45. The Fine–Gray and cause-specific approaches produced closely similar 45-day predictions. The cause-specific model had an AUC of 0.822 (95% CI, 0.798–0.846), compared with 0.821 (95% CI, 0.796–0.845) for the Fine–Gray model. Brier scores were 0.1224 and 0.1224, respectively. Mean predicted mortality was 19.81% with the cause-specific model and 19.95% with Fine–Gray, compared with an observed 45-day mortality of 20.04%. The corresponding observed-to-expected ratios were 1.011 and 1.004, and calibration slopes were 0.975 and 1.008. The paired difference in AUC between Fine–Gray and the cause-specific model was −0.0012 (bootstrap 95% CI, −0.0040 to 0.0017), and the difference in Brier score was 0.00003 (95% CI, −0.00099 to 0.00111). Patient-level predicted risks were highly concordant (Pearson r = 0.993; Spearman ρ = 0.997), with a mean absolute difference of 0.016. The administratively 45-day-restricted Fine–Gray sensitivity analysis gave an AUC of 0.823, Brier score of 0.1217, observed-to-expected ratio of 1.006, and calibration slope of 0.985. Regression effect estimates differed in magnitude, as expected from the different estimands. For example, age per 10-year increase had a cause-specific HR of 1.859 (95% CI, 1.678–2.061) and a Fine–Gray SHR of 1.771 (95% CI, 1.608–1.951). P/F per 100-mmHg increase had corresponding estimates of 0.672 (95% CI, 0.612–0.738) and 0.592 (95% CI, 0.540–0.649). These differences should not be interpreted as disagreement between models because the cause-specific HR and subdistribution HR quantify different aspects of the competing-risk process.

### 3.5. Historical Scores and Exploratory Mexican Evaluation

**Figure 5:**
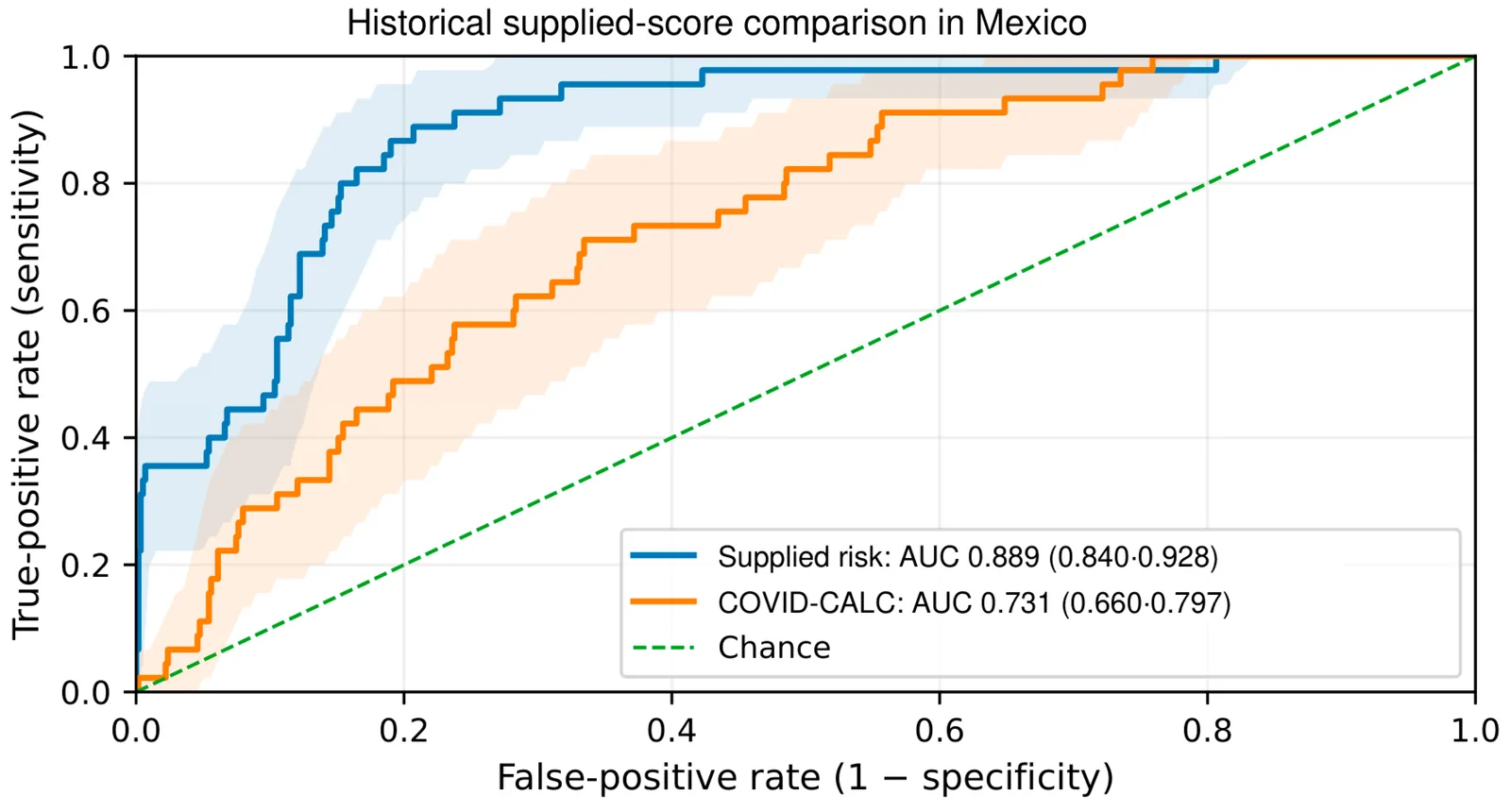
Historical supplied-score ROC comparison in 634 Mexican records, including 45 deaths. Curves and pointwise bootstrap bands retain the original supplied-score analysis; descriptive labels have been revised to avoid an unsupported validation claim. The endpoint is recorded hospital mortality with an unverified prediction horizon. The plot does not evaluate newly generated predictions from the expanded Barcelona models.

The separate historical score file contained 45 deaths among 634 records (7.1%). Supplied Barcelona-labelled scores had AUC 0.889 (95% CI, 0.840–0.928), compared with 0.731 (0.660–0.797) for supplied COVID-CALC scores. The paired AUC difference was 0.157 (0.080–0.234; DeLong *p* < 0.001). AP was 0.488 versus 0.156 and Brier score 0.049 versus 0.168 (Table 9 and Figure 5). Paired AP and Brier differences were 0.332 (0.190–0.461) and −0.119 (−0.139 to −0.098). These comparisons describe the supplied scores only.

**Figure 6:**
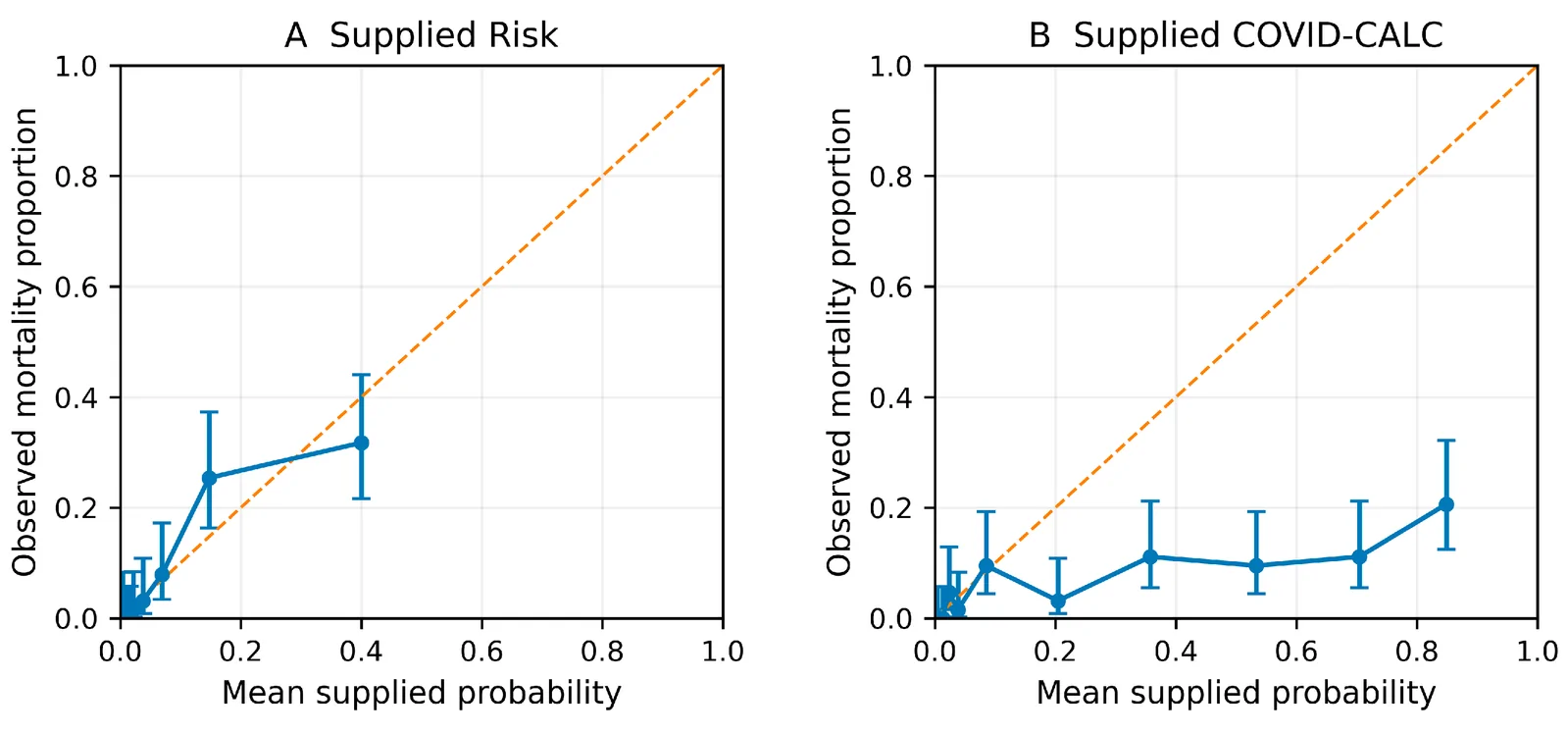
Calibration of historical supplied probabilities in 634 Mexican records: (**A**) supplied risk labelled as the Barcelona score and (**B**) supplied COVID-CALC score. Points summarize risk groups with Wilson 95% intervals; the diagonal indicates ideal calibration. Prediction provenance, prior recalibration and the common horizon are unverified, so these plots cannot establish calibration of the revised Barcelona models.

Mean supplied probability was 7.1% for the Barcelona-labelled scores and 28.0% for COVID-CALC. The former had calibration intercept approximately zero and slope approximately one, whereas the latter had slope 0.296 (Figure 6). The available scores do not establish the prediction-generating model, prior recalibration status or a common 45-day horizon. These numerical results cannot be assigned to either model evaluated below or used as evidence of superiority at the target horizon.

Because COVID-CALC reports 7-, 14-, and 21-day mortality probabilities, a post hoc comparative sensitivity analysis harmonized both models to a 21-day horizon in the Mexico cohort. Death by day 21 was reconstructed from the recorded time-to-death information. The COVID-CALC column was treated as the 21-day app output. The archived fixed Barcelona score originated from a 30-day prediction; a 21-day probability was inferred using a Barcelona-only mapping between exact 21- and 30-day model predictions (logit[F21] = −0.2112 + 0.9638 × logit[F30]; R^2^ = 0.998; MAE = 0.0054). Both scores were then evaluated against the identical 21-day endpoint without refitting or recalibration in Mexico. At the harmonized 21-day horizon in the 634-patient Mexican cohort, the inferred BCN score showed an AUC of 0.868 (95% CI, 0.808–0.917) compared with 0.708 (95% CI, 0.627–0.786) for COVID-CALC. Corresponding Brier scores were 0.042 and 0.170, respectively; these results describe cohort-specific comparative performance at a common 21-day horizon and should not be interpreted as evidence of general model superiority.

In the workbook, 202 hospitalized records were identified. Sequential exclusions comprised invalid admission year (n = 1), missing predictors (11), saturation outside the derivation rule (3), non-positive derived P/F (6), S/F below minimum saturation (1), conflicting LDH across sheets (8) and unresolved day-45 death classification (1). The source-eligible set contained 171 patients with 12 reconstructed deaths by day 45 (7.0%). Excluding the single extreme-LDH record reproduced the earlier 170-person comparison set with 11 deaths (6.5%). These cohorts are separate from the historical 634 scores.

For descriptive LOS, 200 records remained after two evident duration/date errors were excluded. Median stored LOS was 10 days (IQR, 7–16.25), and 194/200 (97.0%) were recorded as ≤45 days; 187/200 (93.5%) were ≤28 days and 189/200 (94.5%) were ≤30 days. This LOS set is distinct from the 200 records with a classifiable day-45 endpoint before predictor exclusions. These summaries show that 45 days covers most retained recorded stays; they do not verify a prospectively selected horizon or the LOS of the unlinked 634-score cohort.

**Figure 7:**
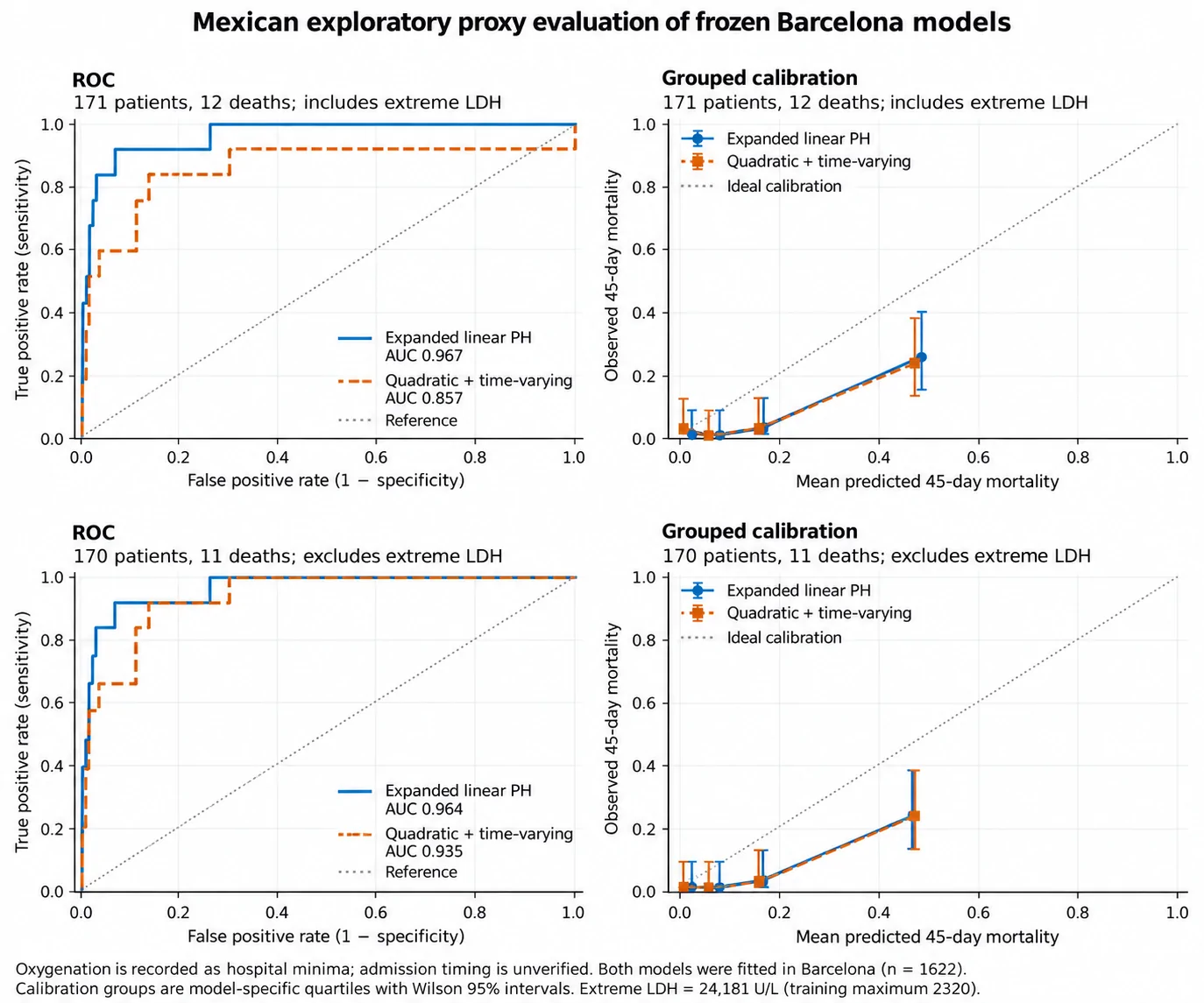
Exploratory Mexican proxy evaluation of the frozen expanded linear and QTV models. Top: 171 patients with 12 reconstructed day-45 deaths, including the extreme LDH record. Bottom: 170 patients with 11 deaths after excluding that record. Left: ROC curves. Right: model-specific quartile calibration points with Wilson 95% intervals. Oxygenation represents hospital minima, with admission timing unverified. These plots do not establish admission-time external validation.

In the 171-person proxy evaluation, the expanded linear reference model had AUC 0.967 (95% CI, 0.916–0.997) and Brier 0.0521 (0.0378–0.0684), compared with 0.857 (0.662–0.976) and 0.0641 (0.0444–0.0870) for QTV (Table 10; Figure 7). Mean predicted risks were 19.3% and 17.7%, respectively, against 7.0% observed. QTV-minus-linear differences were −0.1095 for AUC (95% CI, −0.3066 to −0.0084) and +0.01205 for Brier (0.00112–0.02790).

The extreme record had LDH 24,181 U/L, compared with a Barcelona maximum of 2320 U/L. For this death case, QTV extrapolated to an effectively zero probability and the linear reference to nearly one. After excluding it, AUC was 0.964 (0.907–0.997) for the linear model and 0.935 (0.868–0.984) for QTV; Brier scores were 0.0524 and 0.0586. Mean QTV risk was 17.8% against 6.5% observed, giving O/E 0.364. Paired differences were −0.0286 for AUC (−0.0633 to −0.0039) and +0.00624 for Brier (−0.00025 to 0.01434). No source value was corrected on the basis of these results.

Alternative endpoint, LDH-source and oxygenation-unit scenarios retained overprediction and lower QTV discrimination (Table 11). At 28 and 30 days the same 171-person set had 12 events; the alternative horizons were descriptive sensitivities, not selected replacements. The day-0/1 hemogram subset had 92 patients and only two deaths, so its estimates are descriptive and do not establish baseline oxygenation.

### 3.6. Temporal Validation Across Pandemic Waves

Of the 1622 patients in the oxygenation-expanded Barcelona analysis, 1618 had admission dates within the prespecified Catalonia wave periods. Wave 1 included 1071 patients with 215 deaths by day 45, Wave 2 included 181 patients with 38 deaths, and Wave 3 included 366 patients with 71 deaths. Observed 45-day mortality was 20.1%, 21.0%, and 19.4%, respectively.

Discrimination remained broadly similar across successive waves. AUC was 0.837 (95% CI, 0.808–0.866) in Wave 1, 0.825 (95% CI, 0.751–0.894) in Wave 2, and 0.789 (95% CI, 0.732–0.840) in Wave 3. Average precision was 0.575, 0.611, and 0.506, respectively. The maximum between-wave difference in AUC was 0.049, and the outcome-stratified permutation test did not demonstrate significant AUC heterogeneity (*p* = 0.395).

Calibration showed greater temporal variation. Mean predicted 45-day mortality increased from 18.1% in Wave 1 to 22.5% in Wave 2 and 23.5% in Wave 3, while observed mortality remained approximately 20%. The corresponding observed-to-expected ratios were 1.107, 0.935, and 0.827. Calibration-in-the-large values were +0.168, −0.116, and −0.317, and calibration slopes were 1.038, 1.020, and 0.884, respectively. Calibration intercepts differed significantly across waves (likelihood-ratio *p* = 0.016), whereas there was no evidence of significant heterogeneity in calibration slopes (*p* = 0.589).

The chronological hold-out analysis produced a similar pattern. A model fitted exclusively in Wave 1 and applied to Wave 2 achieved an AUC of 0.826 (95% CI, 0.750–0.898), Brier score of 0.122, O/E ratio of 0.860, and calibration slope of 0.981. When the Wave-1 model was transported directly to Wave 3, AUC remained 0.782 (95% CI, 0.721–0.841), but mean predicted risk was 25.5% compared with observed mortality of 19.4%, corresponding to an O/E ratio of 0.760 and calibration slope of 0.796.

When the model was instead trained on Waves 1 and 2 combined and evaluated on Wave 3, AUC was 0.781 (95% CI, 0.725–0.835), Brier score 0.135, O/E ratio 0.781, and calibration slope 0.826. Thus, adding Wave-2 data before temporal transport did not eliminate the later calibration drift.

Clinical management also changed markedly over calendar time. The proportion of patients receiving corticosteroids increased from 45.9% in Wave 1 to 83.4% in Wave 2 and 91.3% in Wave 3. Tocilizumab use was 23.1%, 16.0%, and 20.8%, respectively. These variables were not part of the admission prediction model and these descriptive differences should not be interpreted causally.

**Figure 8:**
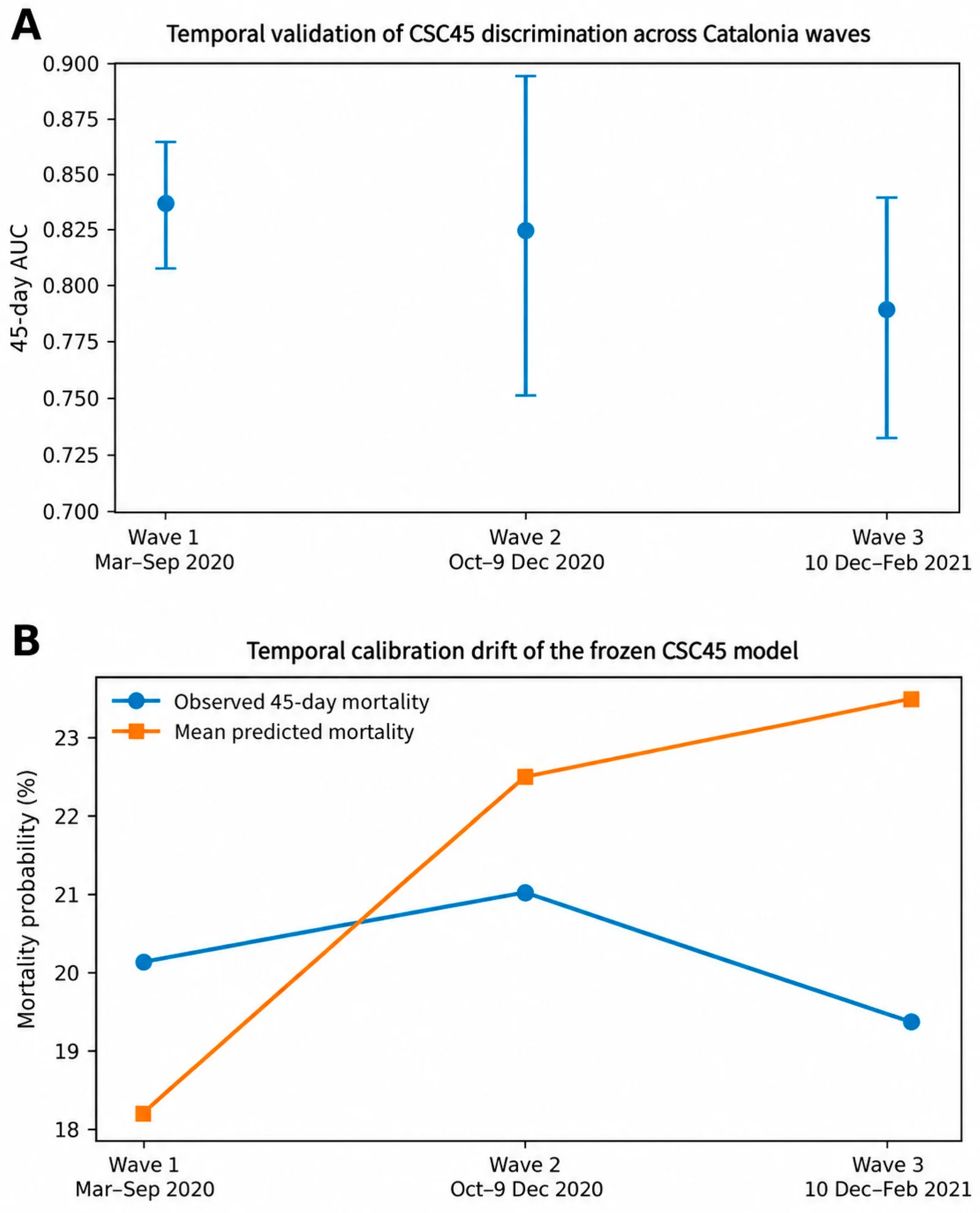
Temporal internal validation of the CSC45 model across successive COVID-19 waves in Barcelona. (**A**) Wave-specific 45-day AUCs were 0.837, 0.825, and 0.789 for Waves 1–3, respectively, with no significant discrimination heterogeneity (*p* = 0.395). (**B**) Observed versus mean predicted 45-day mortality demonstrated temporal calibration drift, with observed-to-expected ratios of 1.107, 0.935, and 0.827 across Waves 1–3. Discrimination remained relatively stable, whereas absolute-risk calibration deteriorated over time.

The sensitivity analysis using narrower Bellvitge metropolitan wave windows produced similar results. AUCs were 0.828, 0.824, and 0.793 and O/E ratios were 1.190, 0.942, and 0.838 for Waves 1, 2, and 3, respectively (Figure 8).

### 3.7. Decision Curve Analysis Results

**Figure 9:**
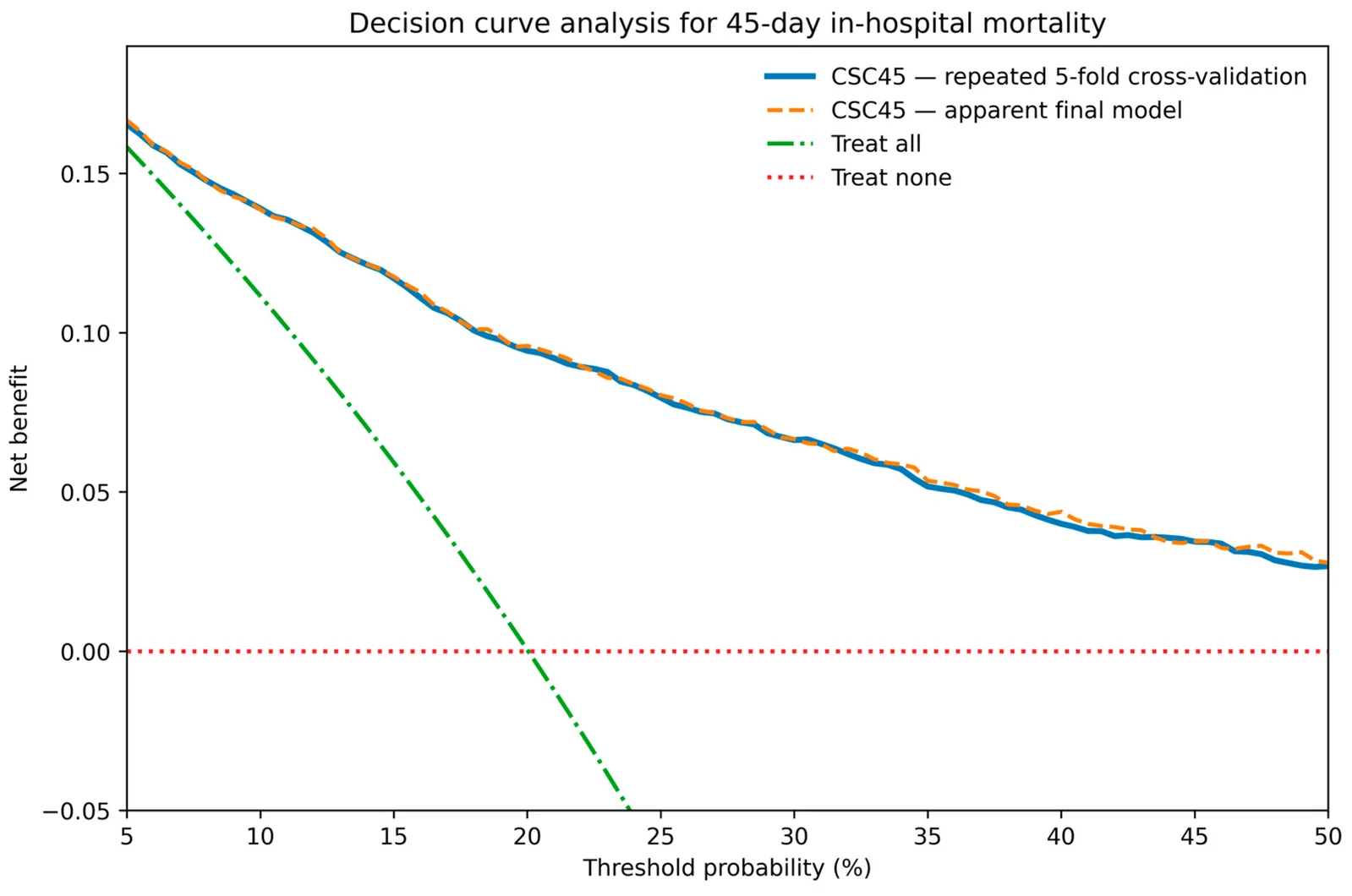
Decision curve analysis of the CSC45 model for 45-day in-hospital mortality. The cross-validated and apparent CSC45 curves showed greater net benefit than treat-all and treat-none strategies across threshold probabilities of 5–50%. Threshold probabilities represent relative weighting of false-positive versus false-negative decisions and should not be interpreted as recommended clinical-action thresholds.

Decision curve analysis indicated a positive net benefit for the CSC45 model across the evaluated threshold probabilities of 5–50% (Figure 9). More importantly, the repeated 5-fold cross-validated decision curve remained above both the treat-all and treat-none strategies throughout this range.

Cross-validated net benefit was 0.165 at a 5% threshold, 0.139 at 10%, 0.117 at 15%, 0.094 at 20%, 0.080 at 25%, 0.066 at 30%, 0.040 at 40%, and 0.027 at 50%. At the 20% threshold, for example, the model provided a net benefit of 0.094 compared with 0.0005 for the treat-all strategy and 0 for the treat-none strategy. At thresholds above approximately the observed mortality prevalence, the treat-all strategy became unfavorable, whereas model-guided classification retained positive net benefit.

The apparent final-model decision curve closely followed the cross-validated curve. At a 20% threshold, apparent and cross-validated net benefits were 0.096 and 0.094, respectively. Pointwise bootstrap intervals for the incremental cross-validated net benefit relative to the better default strategy remained above zero across the evaluated 5–50% range. These results support potential decision value beyond default all-or-none strategies, while not defining a specific clinical action or optimal threshold.

### 3.8. Offline Calculator

The calculator implements the expanded linear reference model and reproduces its fitted probabilities of death, discharge alive and continued hospitalization through day 45 (Supplement S4). Across the original 20 reference profiles, maximum absolute browser-to-reference difference was 1.11 × 10^−16^. Missing inputs prevented calculation; modifying an input invalidated the prior result, and saturation above 97% could not enter the Rice approximation. The interface remains a research implementation. The quadratic plus time-varying candidate has not replaced its parameters or prediction algorithm.

**Figure 10:**
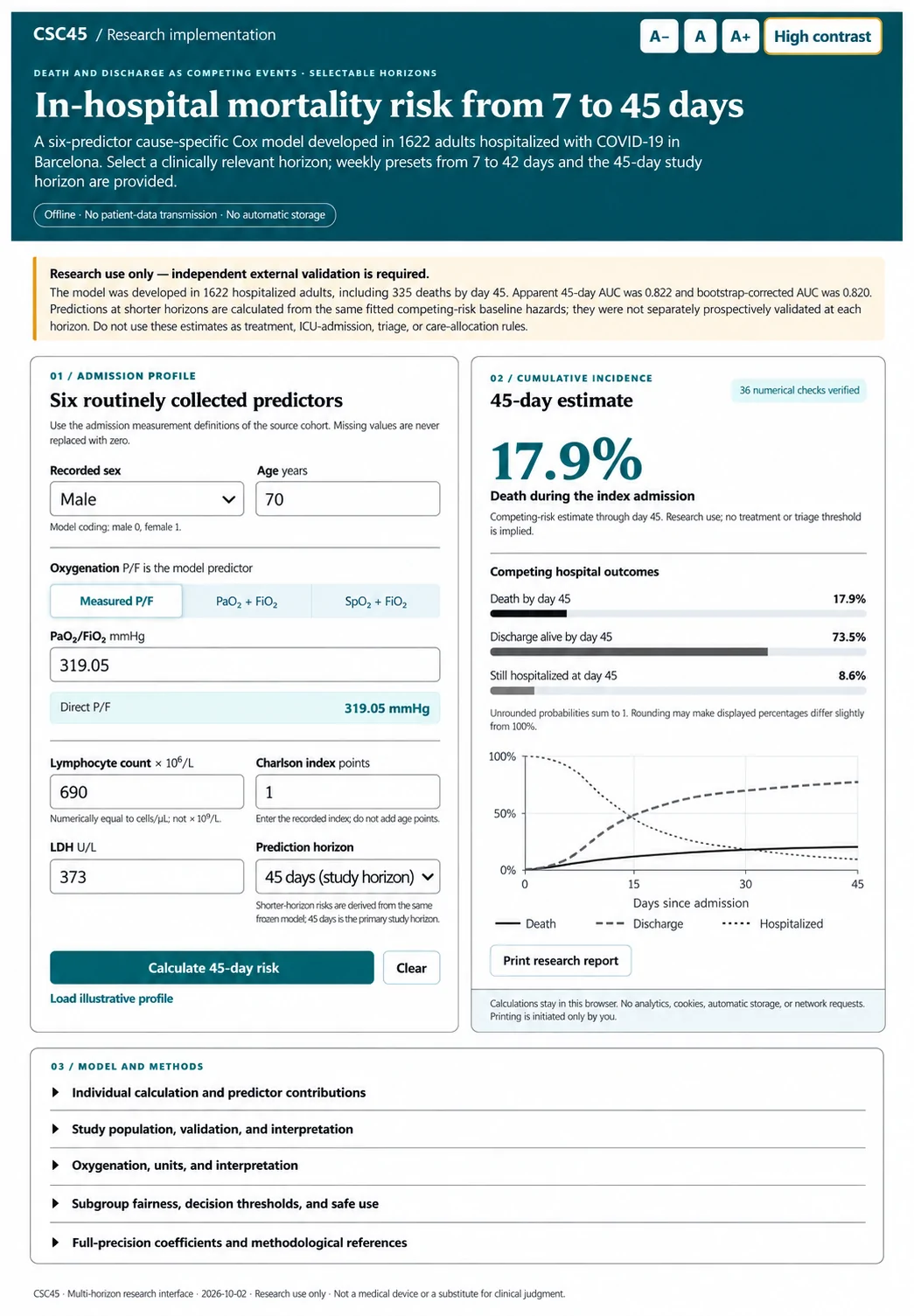
Offline calculator for the oxygenation-expanded linear reference model. The displayed patient profile is hypothetical, and its P/F is entered directly. The three hospital-state probabilities sum to one before rounding. The screenshot does not implement the quadratic plus time-varying candidate. Neither historical supplied scores nor the Mexican proxy evaluation establishes admission-time clinical validity.

Figure 10 shows a hypothetical 70-year-old man with directly entered P/F 319.05 mmHg, lymphocytes 690 × 10^6^/L, CCI 1 and LDH 373 U/L. The reference-model probabilities are 17.9% for death, 73.5% for discharge alive and 8.6% for continued hospitalization at day 45. The interface also supports an explicitly labelled saturation-based route, states the research-use limitation and supplies no treatment or triage threshold. Numerical agreement verifies implementation, not clinical validity or suitability of this profile for decision-making.

## 4. Discussion

The expanded Barcelona analysis included 1622 adults and retained 588 additional patients through explicitly defined oxygenation derivation. The linear reference model had bootstrap-corrected AUC 0.820 and Brier 0.123. Reanalysis confirmed substantial PH and linearity departures rather than resolving them through a larger sample. Adding quadratic and time-varying effects improved cross-validated AUC and Brier score, but calibration slope moved farther from one, and residual discharge-model restrictions remained. In the Mexican workbook, both frozen models overpredicted average mortality and the combined extension had lower AUC. The findings therefore support a transparent comparison of model specifications, rather than an externally validated clinical prediction The entire prognostic TRIPOD-AI details were provided in Supplement S5.

The treatment of oxygenation was important both statistically and clinically. Using only measured arterial P/F would have limited analysis to 1034 patients. Applying a defined saturation-based derivation before assessing predictor completeness allowed inclusion of an additional 588 patients. Their lower mortality and LDH concentrations suggest that restricting analysis to arterial measurements would have produced a cohort with a different severity distribution. Missing arterial P/F should therefore not be equated with an absence of usable oxygenation information when contemporaneous saturation and oxygen-delivery measurements exist.

The broader inclusion does not make the two oxygenation routes interchangeable. The Rice equation was derived in acute lung injury and acute respiratory distress syndrome and is an approximation rather than a direct arterial measurement [13]. FiO_2_ estimates under low-flow oxygen may vary with breathing pattern and oxygen delivery. Saturation also provides less information about PaO_2_ near the upper plateau of the dissociation curve. Restricting derivation to SpO_2_ ≤ 97% avoids extrapolation beyond the saturation criterion used for that relationship, but does not eliminate measurement error. The relatively high P/F values among saturation-derived observations should be interpreted in that context.

The decision-curve analysis complements discrimination and calibration by examining the consequences of threshold-based use of the predicted probabilities. The internally cross-validated curve showed higher net benefit than either an all-patient or no-patient strategy over the evaluated 5–50% threshold range. However, clinical utility is decision-specific: no therapeutic intervention or resource-allocation rule was linked prospectively to these thresholds. The findings therefore indicate potential usefulness for risk-informed decision support rather than evidence that calculator-guided care improves outcomes. External decision-curve evaluation and prospective impact assessment are required before clinical implementation.

The cause-specific formulation represents both death and discharge, and mortality probability depends on both fitted hazards. Associations in Table 4 are working summaries of the linear reference model. In the flexible extension, a predictor contrast depends on its starting value and time since admission. Discharge practices and lengths of stay can change in-hospital risk even when death-specific associations appear similar [6,7]. The diagnostic evidence of residual nonlinearity in the discharge model is therefore relevant to mortality prediction, not merely to a secondary endpoint.

The repeated cross-validation comparison directly addresses the assumption concerns. Quadratic terms produced a larger improvement in AUC and Brier score than time interactions alone, while the combined model had calibration slope 0.830 compared with 0.945 for the reference. Small average gains do not imply interchangeable predictions: the linear time-varying extension changed individual apparent risk by a median 0.80 percentage points and up to 7.71 points. The simple model is retained as a reproducible reference, not because its PH or linearity assumptions hold. The unpenalized combined candidate is reported as a diagnostic extension because its remaining discharge-model misspecification, calibration and extrapolation behavior do not support automatic replacement. Restricted nonlinear functions with appropriate shrinkage warrant Barcelona-only development and full internal validation before a primary model is selected.

The Mexican analyses have different evidential roles. The historical 634 scores support only a descriptive comparison of supplied probabilities, because provenance, patient linkage and the horizon are unverified. The workbook allows actual frozen-model scoring but contains minimum rather than verified admission oxygenation. Later deterioration can introduce information unavailable at admission and distort apparent discrimination. Consequently, even an AUC near 0.97 cannot establish admission-time validity. The combined model’s mean predicted mortality of 17.8% against 6.5% observed in the 170-person sensitivity illustrates why discrimination and calibration must be considered together [32]. Neither source supports a horizon-matched clinical superiority claim over COVID-CALC.

The comparison with Fine–Gray clarifies the rationale for the primary cause-specific framework. Fine–Gray has a particularly direct relationship with the cumulative incidence function and is therefore useful when the principal inferential target is how covariates are associated with the probability of the event of interest over time. The cause-specific approach instead describes transition rates among patients who remain event-free and permits the death and recovery/discharge processes to be examined separately. That distinction was important for the present study. Clinically, a hospitalized patient’s absolute probability of death depends not only on the instantaneous hazard of deterioration and death but also on the competing rate of recovery and discharge. The primary model therefore estimated both processes and combined them to obtain absolute mortality risk. This representation also permits the calculator to provide mutually interpretable probabilities of death, discharge alive, and continued hospitalization at the prediction horizon. The Fine–Gray sensitivity analysis showed that this modeling choice was not responsible for the observed prognostic performance. Forty-five-day mortality probabilities from the two approaches were highly concordant, and discrimination, Brier score, and average calibration were essentially unchanged. Nevertheless, their regression coefficients should not be compared as if they were estimates of the same effect. A cause-specific hazard ratio describes the relative instantaneous event rate among patients still in the hospitalized/event-free state, whereas a Fine–Gray subdistribution hazard ratio describes the relative subdistribution rate linked to cumulative incidence and incorporates the effect of the competing-event process through its extended risk set. Accordingly, the cause-specific framework was retained as the primary model because it corresponds to the study’s multistate clinical representation of death versus discharge, while the Fine–Gray analysis provides complementary evidence that the 45-day prognostic conclusions are robust to an alternative competing-risk regression estimand.

**Figure 11:**
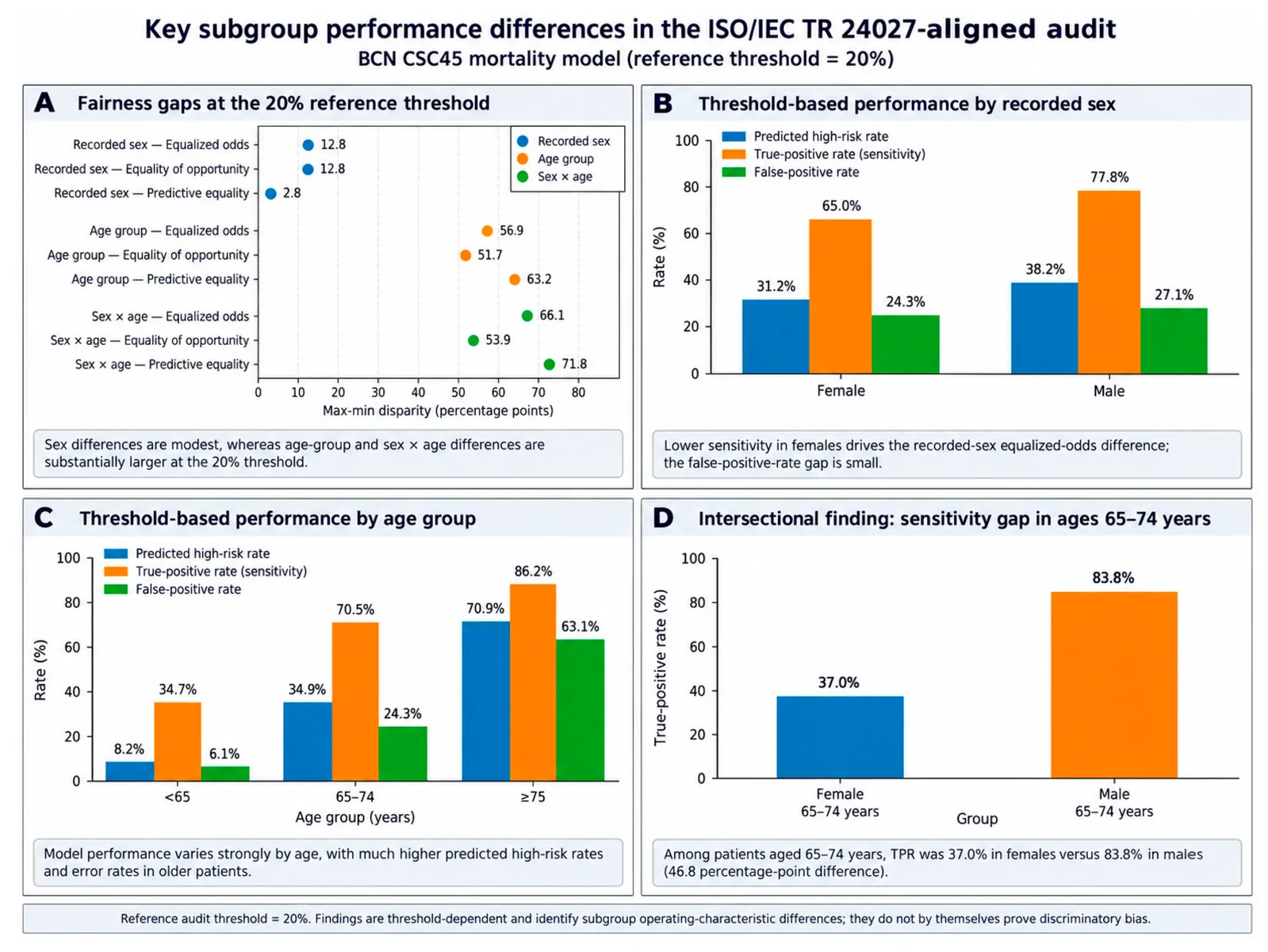
Subgroup performance differences identified in the ISO/IEC TR 24027-aligned bias and fairness audit of the CSC45 model. (**A**) Max–min disparities in threshold-based fairness metrics at the 20% reference risk threshold, showing smaller differences by recorded sex and larger differences across age and sex-by-age subgroups. (**B**) Predicted high-risk rate, true-positive rate, and false-positive rate by recorded sex; the sex-related equalized-odds difference was driven primarily by lower sensitivity in females. (**C**) Corresponding operating characteristics across age groups, demonstrating progressively larger high-risk classification and error rates with increasing age. (**D**) Intersectional analysis among patients aged 65–74 years, showing a TPR of 37.0% in females versus 83.8% in males. The 20% threshold was used as an audit operating point and should not be interpreted as a recommended treatment or triage threshold; the observed differences are threshold dependent and do not by themselves establish discriminatory bias.

A post-hoc subgroup audit aligned with ISO/IEC TR 24027 showed that threshold-based performance was not fully uniform across demographic groups (Figure 11). At the 20% reference threshold, sensitivity was lower in females than males (65.0% vs. 77.8%), corresponding to a 12.8-percentage-point equality-of-opportunity gap (permutation *p* = 0.0196; FDR q = 0.0232), whereas false-positive rates were similar (24.3% vs. 27.1%; predictive-equality gap 2.8 percentage points; *p* = 0.276). The sex-related equalized-odds finding was therefore driven mainly by a difference in sensitivity rather than false-positive classification (detailed analysis in Supplement S3).

Larger differences were observed across age groups and in the intersectional sex-by-age analysis (Figure 11). High-risk classification rates increased from 8.2% in patients aged <65 years to 34.9% in those aged 65–74 years and 70.9% in those aged ≥75 years, with corresponding TPRs of 34.7%, 70.5%, and 86.2%. The most prominent localized finding occurred among patients aged 65–74 years, in whom TPR was 37.0% in females versus 83.8% in males. These results should be interpreted as subgroup operating-characteristic differences rather than evidence of discriminatory bias, because age is a clinically relevant prognostic factor, outcome prevalence differs across subgroups, findings are threshold dependent, and no prospective fairness acceptance margin was prespecified. External subgroup validation and prospective definition of clinically acceptable fairness margins are therefore required before deployment.

Several limitations require emphasis. First, 655 eligible Barcelona patients were excluded from the primary model because of incomplete predictors and differed from included patients in age, LDH, and mortality. However, multiple imputation of all 2277 eligible patients produced very similar performance to the primary analysis (AUC, 0.830 vs. 0.822; Brier score, 0.113 vs. 0.122; O/E, 0.994 vs. 1.011; calibration slope, 0.962 vs. 0.975), and predictions within the original cohort were highly concordant (r = 0.997). Thus, complete-case exclusion was unlikely to materially drive the results under a missing-at-random assumption, although residual selection bias cannot be excluded. The Rice-derived P/F subgroup also differed markedly from patients with direct arterial P/F, including lower mortality and LDH and higher P/F. Nevertheless, discrimination was similar between measured and derived groups (AUC, 0.818 vs. 0.792; *p* = 0.382), calibration did not differ significantly, and a measured-P/F-only refit produced nearly identical overall performance and risks (r = 0.993). A borderline P/F-by-route interaction for mortality (*p* = 0.0477) indicates that measurement or residual case-mix effects remain possible. Therefore, these analyses support prognostic robustness but do not establish physiologic equivalence of Rice-derived and directly measured P/F.

Second, although temporal internal validation showed that discrimination remained reasonably stable across successive pandemic waves, absolute-risk calibration changed over time. Wave-specific AUCs ranged from 0.789 to 0.837 without significant heterogeneity, whereas O/E ratios declined from 1.107 in Wave 1 to 0.827 in Wave 3, indicating increasing overprediction in the later period. Chronological hold-out analyses showed the same pattern, with preserved but lower discrimination in Wave 3 and deterioration in calibration. These findings are consistent with changes in case mix, treatment, hospital practice, immunity, and viral epidemiology over the study period and indicate that recalibration or model updating should be considered before applying the model to later clinical settings

Fourth, uncertainty in FiO_2_ estimation and saturation-derived P/F was not formally propagated through model coefficients or individual risk estimates, although the oxygenation-route sensitivity analyses showed broadly stable discrimination and calibration. In the Mexican evaluations, lymphocyte units, CCI compatibility, measurement timing, and outcome reconstruction remained partly assumption-dependent, and ambiguous records required exclusion or sensitivity analyses. Fifth, the Mexican workbook analyses contained only 11–12 target events, resulting in wide confidence intervals and unstable calibration estimates. Cross-validation and bootstrap procedures quantify uncertainty conditional on the specified analyses but do not capture all uncertainty arising from post-hoc model development, data reconstruction, or source-data limitations.

Finally, numerical calculator verification does not establish usability, clinical benefit, fairness or safety for treatment decisions.

The practical contribution is a fully specified competing-risk reference model, empirical diagnostics, executed flexible-model comparisons and an exploratory transport assessment. Coefficients, centering and scaling constants, baseline hazards and reference calculations distinguish the expanded reference model from the 17-coefficient-per-cause candidate. Further development should control nonlinear extrapolation and overfitting, assess remaining time dependence in both causes and use compatible admission predictors with a verified common endpoint in a genuinely independent validation cohort. Any future tuning after examining Mexican performance must be distinguished from untouched external validation. The current calculator remains a research implementation of the reference model.

## 5. Conclusions

Oxygenation derivation increased Barcelona cohort retention from 1034 to 1622 patients, while multiple-imputation analysis of all 2277 eligible patients yielded closely similar discrimination and calibration, reducing concern that complete-case exclusion materially determined the findings. Sensitivity analyses also showed that inclusion of Rice-derived P/F did not materially alter overall predictive performance or individual risk estimates, although the two oxygenation groups differed substantially in case mix and the Rice relationship was not directly physiologically validated in COVID-19. Results were similarly robust to use of a Fine–Gray competing-risk formulation. The linear reference model showed useful discrimination, but proportional-hazards and linearity departures remained; more flexible extensions improved some internal metrics without resolving all model-assumption or transportability concerns. Temporal validation showed relatively preserved discrimination across pandemic waves but meaningful drift in absolute-risk calibration, indicating a potential need for recalibration in later clinical settings. Decision-curve analysis suggested potential net benefit across a clinically relevant range of thresholds, whereas subgroup analyses identified threshold-dependent performance differences that warrant continued fairness monitoring. Exploratory Mexican analyses remain limited by endpoint and source-data uncertainties and do not constitute validation of the updated 1622-patient model. Overall, the findings support prognostic robustness across several sensitivity analyses, but independent contemporary validation with harmonized outcomes, verified predictors, subgroup assessment, and prospective evaluation of clinical impact remains necessary before clinical implementation.

## Figures and Tables

**Table 1:** Selected COVID-19 mortality prediction approaches and their relevance to the present study.

Study/Model	Population or Evaluation	Approach	Relevance
4C dynamic reassessment [10]	6373 hospitalized patients; repeated assessment	Repeated bedside score	Distinguishes admission from later prediction landmarks.
ABC_2_-SPH [2]	Development and temporal/external validation	Admission clinical score	Routine measurements; validation across settings.
COVID-CALC [9]	1810 development and 381 validation patients	Fine–Gray competing-risk model	Original app reports risks at 7, 14, and 21 days.
ALPACA [11]	Development and validation in the southern United States	Parsimonious multivariable model	Illustrates geographically defined validation.
CZ-COVID-19 [12]	388 development and 1357 validation patients	Seven-variable logistic score	Evaluates performance after transport.
Current study	1622 Barcelona patients; separate Mexican workbook and 634 supplied-score records	Reference cause-specific Cox model; quadratic and time-varying extensions; calculator	Internal validation and diagnostic comparisons; exploratory external proxy evaluation

**Table 2:** Study populations, outcomes, and ethical approvals.

Characteristic	Barcelona	Monterrey, Mexico
Design and setting	Retrospective hospital cohort; Bellvitge University Hospital, Barcelona, Spain.	Independent SCOURGE clinical workbook and a separate historical score file; exploratory evaluation.
Study period	Admissions from March 2020 to February 2021.	Parent Latin-American SCOURGE recruitment: March 2020–July 2021.
Population	2285 patients assessed; 2277 eligible adults; 1622 included after oxygenation derivation and predictor-completeness criteria.	227 identified workbook records; 202 hospitalized; 171 source-eligible for scoring (170 without extreme LDH). Separate historical file: 634 scores.
Outcome and analytical role	45-day index-admission death; discharge alive as competing event. Reference development, bootstrap validation and repeated CV of extensions.	Workbook: 12 deaths by day 45 among 171 records (11/170 without extreme LDH), conditional on reconstruction. Historical file: 45 binary hospital deaths among 634 scores; horizon unverified.
Ethical approval	Bellvitge University Hospital research ethics committee; PR128/20.	Tecnológico de Monterrey School of Medicine Ethics and Research Committee; P000396-GenoCOVID-CEIC-CR002. COFEPRIS authorization 203301410A0265/2020; Galician Ethical Committee reference 2020/197.
Consent	Individual informed consent was waived for the retrospective Barcelona study.	Informed consent was obtained in the parent SCOURGE study.

SCOURGE, Spanish Coalition to Unlock Research on Host Genetics on COVID-19; COFEPRIS, Federal Commission for the Protection against Sanitary Risks. Cohort descriptions and parent-study approvals are supported by the original reports [16,17].

**Table 3:** Characteristics of the Barcelona analysis population, oxygenation subgroups, and eligible patients not included because of remaining predictor incompleteness.

Characteristic	Analysis Cohort (n = 1622)	Measured P/F (n = 1034)	Derived P/F (n = 588)	Not Included (n = 655)
Female sex, n (%)	597 (36.8)	344 (33.3)	253 (43.0)	265 (40.5)
Age, years	68.8 [56.8–77.7]	68.6 [57.0–77.4]	69.2 [56.5–78.4]	63.9 [53.0–75.2]
P/F, mmHg	338.1 [271.4–462.2]	295.2 [233.3–342.9]	465.2 [451.0–473.7]	352.4 [261.9–468.0]
Lymphocytes, ×10^6^/L	890 [633–1223]	870 [603–1203]	910 [683–1283]	1000 [723–1343]
CCI, points	0 [0–2]	1 [0–2]	0 [0–1]	0 [0–2]
LDH, U/L	325 [264–421]	348 [276–455]	292.5 [242–360.3]	258 [220–321]
Hospital stay, days	10 [6–21]	11 [7–22]	9 [6–18]	8 [4–18]
Death by day 45, n (%)	325 (20.0)	256 (24.8)	69 (11.7)	85 (13.0)
Discharge by day 45, n (%)	1204 (74.2)	718 (69.4)	486 (82.7)	529 (80.8)
Hospitalized at day 45, n (%)	93 (5.7)	60 (5.8)	33 (5.6)	41 (6.3)

Continuous variables are median [IQR]. P/F, PaO_2_/FiO_2_ ratio; CCI, Charlson Comorbidity Index; LDH, lactate dehydrogenase. Among the 655 patients not included, P/F, lymphocytes, and LDH were available for 215, 637, and 386 patients, respectively; age, sex, CCI, and outcomes were available for all 655. The two oxygenation subgroups partition the analysis cohort. Percentages may not total 100% because of rounding.

**Table 4:** Working constant-effect summaries from the linear cause-specific reference models in 1622 Barcelona patients.

Predictor Contrast	Death HR (95% CI)	*p* Value	Discharge Alive HR (95% CI)	*p* Value
Female versus male	0.917 (0.724–1.161)	0.470	1.110 (0.989–1.246)	0.076
Age, per 10 years	1.859 (1.678–2.061)	<0.001	0.900 (0.864–0.937)	<0.001
P/F, per 100 mmHg	0.672 (0.612–0.738)	<0.001	1.299 (1.229–1.374)	<0.001
Lymphocytes, per 500 ×10^6^/L	0.986 (0.954–1.019)	0.398	1.003 (0.992–1.015)	0.590
CCI, per point	1.128 (1.066–1.194)	<0.001	0.905 (0.866–0.945)	<0.001
LDH, per 100 U/L	1.091 (1.041–1.143)	<0.001	0.893 (0.855–0.933)	<0.001

Both reference models adjust for all six predictors and use full hospitalization follow-up. Intervals are model-based Wald intervals under the working specification. Significant PH and functional-form departures limit interpretation; these are not coefficients of the quadratic plus time-varying model.

**Table 5:** Internal performance of the expanded linear reference model for 45-day in-hospital death in Barcelona.

Measure	Apparent Estimate (95% CI)	Bootstrap-Corrected Estimate
AUC	0.822 (0.798 to 0.845)	0.820
Average precision	0.551 (0.503 to 0.604)	0.542
Brier score	0.122 (0.115 to 0.130)	0.123
Scaled Brier score	0.236 (0.191 to 0.280)	0.230
Observed-to-expected ratio	1.011 (0.968 to 1.055)	—
Calibration-in-the-large	0.019 (−0.054 to 0.089)	—
Calibration slope	0.975 (0.864 to 1.098)	0.958

Apparent confidence intervals use 2000 outcome-stratified resamples of fixed predictions and are conditional on the fitted model. Optimism correction uses 1000 patient-level bootstrap resamples with both hazards refitted. Corrected values are point estimates, not confidence limits. Ideal calibration-in-the-large is 0; ideal calibration slope and observed-to-expected ratio are 1. Lower Brier scores indicate less error. The event proportion was 0.200.

**Table 6:** Global PH score tests for the linear reference models; all tests have six degrees of freedom.

Analysis	Death χ^2^	Death *p*	Discharge χ^2^	Discharge *p*
Full hospitalization	49.881	4.97 × 10^−9^	109.283	2.88 × 10^−21^
Estimation through day 45	54.159	6.85 × 10^−10^	79.838	3.86 × 10^−15^
Exclude day-zero outcomes	47.401	1.56 × 10^−8^	109.208	2.99 × 10^−21^

Tests use predictor-by-log(1 + time) interactions, adjusted for the reference main effects. Adding quadratic main effects alone also left significant time variation (death *p* = 2.31 × 10^−7^; discharge *p* = 1.39 × 10^−24^; 11 df).

**Table 7:** Mean performance across three repetitions of five-fold cross-validation for 45-day death in Barcelona.

Specification	AUC	Brier Score	Calibration Slope
Linear PH reference	0.8193	0.12343	0.945
Quadratic PH	0.8296	0.12017	0.846
Linear time-varying	0.8223	0.12273	0.946
Quadratic plus time-varying	0.8307	0.11974	0.830

All four models use the same patients and folds, with both hazards fitted only in each training fold. Quadratic models add five squared continuous terms; time-varying models add six log-time interactions. Lower Brier scores are better; ideal slope is 1. These are internal estimates conditional on the fixed candidate specifications.

**Table 8:** Exploratory conditional score tests for additional terms beyond the 17-coefficient-per-cause QTV model.

Additional Term Set	Death *p*	Discharge *p*
Additional log^2^-time interactions (6 df)	0.0535	0.093
Quadratic-by-log-time interactions (5 df)	0.658	0.0176
Additional cubic predictor terms (5 df)	0.45	1.96 × 10^−6^

**Table 9:** Descriptive comparison of historical supplied scores in 634 Mexican records with a binary hospital outcome.

Measure	Supplied Barcelona-Labelled Score (95% CI)	COVID-CALC (95% CI)
AUC	0.889 (0.840–0.928)	0.731 (0.660–0.797)
Average precision	0.488 (0.362–0.623)	0.156 (0.117–0.245)
Brier score	0.049 (0.042–0.056)	0.168 (0.149–0.187)
Mean predicted probability	0.071 (0.062–0.080)	0.280 (0.257–0.302)
Observed-to-expected ratio	1.000 (0.888–1.137)	0.254 (0.235–0.276)
Calibration-in-the-large	0.000 (−0.176 to 0.181)	−2.447 (−2.630 to −2.267)
Calibration slope	1.000 (0.813–1.242)	0.296 (0.164–0.507)

The endpoint is recorded in-hospital mortality, with no verified common prediction horizon. Confidence intervals use 5000 outcome-stratified paired bootstrap resamples. Model provenance, prior updating and linkage to the clinical workbook are unverified. These results are not external validation of the 1622-patient expanded reference fit or its flexible extensions.

**Table 10:** Exploratory 45-day Mexican proxy performance of the frozen expanded Barcelona models, with 95% ordinary-bootstrap intervals.

Patients/Deaths	Metric	Linear Reference	QTV
171/12	AUC	0.967 (0.916–0.997)	0.857 (0.662–0.976)
171/12	Brier	0.0521 (0.0378–0.0684)	0.0641 (0.0444–0.0870)
171/12	Mean predicted risk	19.3% (16.1–22.6)	17.7% (14.5–20.9)
171/12	Observed to expected	0.363 (0.196–0.525)	0.397 (0.210–0.590)
171/12	Calibration in the large	−1.604 (−2.364–−1.086)	−1.468 (−2.297–−0.879)
171/12	Calibration slope	1.984 (1.318–5.311)	0.228 (−0.148–2.177)
170/11	AUC	0.964 (0.907–0.997)	0.935 (0.868–0.984)
170/11	Brier	0.0524 (0.0383–0.0686)	0.0586 (0.0416–0.0780)
170/11	Mean predicted risk	18.8% (15.9–21.9)	17.8% (14.8–21.0)
170/11	Observed to expected	0.344 (0.176–0.506)	0.364 (0.187–0.540)
170/11	Calibration in the large	−1.604 (−2.353–−1.091)	−1.598 (−2.457–−1.016)
170/11	Calibration slope	1.984 (1.322–5.147)	1.341 (0.900–2.668)

The 171-person set includes the extreme LDH record; the 170-person sensitivity excludes it. Both models were developed in 1622 Barcelona patients and used the same integration. Intervals use 5000 paired ordinary patient resamples with fitted models fixed. Finite calibration fits numbered 4976 and 4999 for linear and QTV in n = 171, and 4978 and 5000 in n = 170. Intervals condition on finite estimates and do not capture proxy or endpoint uncertainty. Calibration regressions were not used to recalibrate predictions.

**Table 11:** Point-estimate sensitivities of the exploratory Mexican evaluation.

Scenario	n/Deaths	Linear AUC	QTV AUC	Linear Brier	QTV Brier
Main cohort at day 28	171/12	0.963	0.856	0.0449	0.0563
Main cohort at day 30	171/12	0.963	0.858	0.0458	0.0572
Ambiguous death at 43 days	172/13	0.946	0.840	0.0557	0.0680
Ambiguous death at 48 days	172/12	0.967	0.858	0.0519	0.0639
Conflicting LDH: main sheet	179/12	0.968	0.859	0.0506	0.0622
Conflicting LDH: secondary sheet	179/12	0.969	0.860	0.0504	0.0617
Alternative S/F unit convention	173/12	0.967	0.858	0.0520	0.0637
Hemogram on calendar day 0 or 1	92/2	0.972	0.950	0.0480	0.0503

All scenarios retain the extreme LDH record when otherwise eligible; the hemogram restriction naturally excludes it. Except for the 28- and 30-day rows, the horizon is 45 days. The alternative S/F convention multiplies values between 0 and 5 by 100 as an explicit assumption scenario, not a verified correction. Intervals for the principal 171/170 comparisons are in Table 10; other scenarios are descriptive.

## Data Availability

Individual-level clinical data are subject to institutional approval, confidentiality requirements and applicable data-sharing agreements; requests may be directed to the corresponding author. The reference-model specification and offline implementation are provided in Supplement S1. Supplement S3 supplies the frozen diagnostic-extension parameters, aggregate results, figures and updated reporting map. Individual-level patient data are not included in the public supplements.

## Supplementary Materials

The following supporting information can be downloaded at: https://www.mdpi.com/article/10.3390/diagnostics16203267/s1, Supplementary Note S1: Artificial Intelligence and Machine Learning in Clinical Prognosis: Relevance to the CSC45 Study; Supplement S2: updated reporting map, PH figures, internal model comparisons, frozen QTV parameters and exploratory Mexican evaluation tables and figures. Supplement S3: ISO/IEC TR 24027-Aligned Bias and Fairness Assessment of the BCN CSC45 Mortality Model; Supplement S4: offline CSC45 calculator for the expanded linear reference model. Supplement S5: TRIPOD-AI checklist.

## Abbreviations

AP, average precision; AUC, area under the receiver operating characteristic curve; CCI, Charlson Comorbidity Index; CI, confidence interval; CITL, calibration-in-the-large; CSC, cause-specific Cox; CV, cross-validation; FiO_2_, fraction of inspired oxygen; HR, hazard ratio; IQR, interquartile range; LDH, lactate dehydrogenase; LOS, length of stay; O/E, observed-to-expected ratio; PaO_2_, arterial oxygen partial pressure; P/F, PaO_2_/FiO_2_ ratio; PH, proportional hazards; QTV, quadratic plus time-varying; S/F, SpO_2_/FiO_2_ ratio; SCOURGE, Spanish Coalition to Unlock Research on Host Genetics on COVID-19; SpO_2_, peripheral oxygen saturation.
