# Supplementary material for: Competing Risk Prediction of COVID-19 Hospital Mortality with Model Diagnostics and Exploratory Evaluation in Mexico
Source: Diagnostics (Basel). 2026 Oct 9;16(20):3267. doi: 10.3390/diagnostics16203267 (PMC13703144; doi:10.3390/diagnostics16203267)
Supplement: Supplementary file 1 [file diagnostics-16-03267-s001.zip › Supplement File S4.html]

CSC45 | 7–45-day in-hospital mortality — research calculator
Skip to calculator

JavaScript is required for local calculations. No risk has been calculated.


CSC45  / Research implementation

A−AA+High contrast

Death and discharge as competing events · selectable horizons

# In-hospital mortality risk from 7 to 45 days

A six-predictor cause-specific Cox model developed in 1,622 adults hospitalized with COVID-19 in Barcelona. Select a clinically relevant horizon; weekly presets from 7 to 42 days and the 45-day study horizon are provided.

Offline · No patient-data transmission · No automatic storage


**Research use only — independent external validation is required.**The model was developed in 1,622 hospitalized adults, including 325 deaths by day 45. Apparent 45-day AUC was 0.822 and bootstrap-corrected AUC was 0.820. Predictions at shorter horizons are calculated from the same fitted competing-risk baseline hazards; they were not separately prospectively validated at each horizon. Do not use these estimates as treatment, ICU-admission, triage, or care-allocation rules.

01 / Admission profile

## Six routinely collected predictors

Use the admission measurement definitions of the source cohort. Missing values are never replaced with zero.

Recorded sexSelectMaleFemale

Model coding: male 0, female 1.

Age years

---

Oxygenation P/F is the model predictor

Measured P/FPaO₂ + FiO₂SpO₂ + FiO₂

PaO₂/FiO₂ mmHg

PaO₂ mmHg

FiO₂ percent

SpO₂ percent

FiO₂ percent

 PaO₂ is unavailable; SpO₂ and FiO₂ are from the same assessment. I understand that the Rice conversion is approximate and is not equivalent to an arterial measurement.

SpO₂ above 97%, category-only SpO₂, or unknown FiO₂ are not supported.

Enter oxygenation measurements**—**

---

Lymphocyte count ×10⁶/L

Numerically equal to cells/µL; not ×10⁹/L.

Charlson index points

Enter the recorded index; do not add age points.

LDH U/L

Prediction horizon7 days (1 week)14 days (2 weeks)21 days (3 weeks)28 days (4 weeks)35 days (5 weeks)42 days (6 weeks)45 days (study horizon)

Shorter-horizon risks are derived from the same frozen model; 45 days is the primary study horizon.

Calculate 45-day riskClear

Load illustrative profile

02 / Cumulative incidence

## 45-day estimate

Checking engine…

—

Death during the index admission

Enter a complete profile and calculate. No result is currently available.

### Competing hospital outcomes

Death by day 45**—**

Discharge alive by day 45**—**

Still hospitalized at day 45**—**

Unrounded probabilities sum to 1. Rounding may make displayed percentages differ slightly from 100%.

DeathDischargeHospitalized

Print research report

Calculations stay in this browser. No analytics, cookies, automatic storage, or network requests. Printing is initiated only by you.

No current valid calculation is available. Return to the application, validate the inputs, and calculate before printing.

03 / Model and methods

Individual calculation and predictor contributions

η_k_(X) = Σ_j_ β_kj_(X_j_ − c_j_), k = 1 death, 2 discharge.  
a_kr_(X) = exp[η_k_(X)] ΔH_0k_(u_r_); A_r_ = a_1r_ + a_2r_.  
F_k,r_ = F_k,r−1_ + S_r−1_[1 − exp(−A_r_)] a_kr_/A_r_.  
S_r_ = S_r−1_ exp(−A_r_). When A_r_ = 0, the probabilities are unchanged.

Death linear predictor**—**

Death hazard multiplier**—**

Discharge hazard multiplier**—**

No current calculation.

| Predictor | Input | Center | Death contribution | Discharge contribution |
| --- | --- | --- | --- | --- |

Contributions are to cause-specific log hazards, not additive contributions to absolute death risk. They are not causal effects.

Study population, validation, and interpretation

The Barcelona study included 1,622 adults hospitalized between March 2020 and February 2021. Recorded arterial P/F was available for 1,034 participants; saturation-based derivation provided oxygenation estimates for another 588. The two fitted cause-specific models describe death and discharge alive during the index hospitalization.

By day 45, 325 participants had died, 1,204 had been discharged alive, and 93 remained hospitalized. The prediction target is in-hospital death during the index admission, not all-cause death after discharge. The app can display cumulative incidence at earlier horizons using the model's daily baseline-hazard grid, but those earlier-horizon estimates were not separately prospectively validated.

Internal validation used 1,000 patient-level bootstrap resamples, with both cause-specific models refitted in every resample. The optimism-corrected 45-day AUC was 0.820, Brier score 0.123, and calibration slope 0.958. Temporal validation across three Catalonia pandemic waves showed relatively preserved discrimination (AUC 0.837, 0.825, and 0.789) but changing absolute calibration (O/E 1.107, 0.935, and 0.827), including overprediction in the later wave. Therefore, recalibration may be required in later clinical settings.

Multiple-imputation sensitivity analysis in all 2,277 eligible Barcelona patients produced results close to the primary analysis (AUC 0.830; Brier 0.113; O/E 0.994; calibration slope 0.962), reducing but not eliminating concern about complete-case selection under a missing-at-random assumption. The app itself does not impute an individual's missing predictor: all six inputs are required.

A Fine–Gray competing-risk sensitivity analysis produced very similar 45-day discrimination and calibration to the cause-specific Cox framework. The calculator retains the cause-specific model because it explicitly represents both death and discharge hazards.

The model assumes linear covariate effects and proportional cause-specific hazards. Diagnostic analyses indicated departures from these assumptions. In addition, saturation-derived oxygenation and changes in treatment, variants, immunity, admission practice, and discharge practice limit generalizability.

The calculation combines the two non-negative hazard increments by exponential integration. Death, discharge, and remaining-hospitalization probabilities are non-negative and sum to one; no clipping, renormalization, or early stopping is used. Numerical agreement is checked against stored reference profiles. Numerical agreement does not demonstrate clinical benefit.

Three same-day outcomes were included in model development. Omitting those admissions produced similar discrimination. Measurements must describe the admission assessment; do not substitute the worst value observed later during hospitalization.

Oxygenation, units, and interpretation

P/F = PaO₂/FiO₂, with FiO₂ expressed as a fraction. This interface accepts FiO₂ in percent and divides it by 100. When PaO₂ is unavailable and SpO₂ ≤97%, the optional Rice estimate is P/F = [(SpO₂/FiO₂) − 64]/0.84, with SpO₂ entered in percentage units. It is not equivalent to a directly measured arterial P/F, and directly measured arterial oxygenation is preferred when available.

Model development used recorded arterial P/F when available and saturation-derived P/F for 588 participants with otherwise complete predictors. In sensitivity analysis, the frozen model had AUC 0.818 in the measured-P/F subgroup and 0.792 in the Rice-derived subgroup (difference −0.025; p=0.382), with no significant calibration-intercept or calibration-slope heterogeneity. However, the two groups differed strongly in case mix and a borderline P/F-by-route interaction was observed for the death hazard. These analyses support prognostic robustness, not physiologic interchangeability or direct validation of the Rice relationship in COVID-19.

Enter the non-age-adjusted Charlson comorbidity score. Age is included separately. Laboratory units must match the units shown above.

No low-, medium-, or high-risk categories, treatment thresholds, or individual confidence intervals are implied. The tool estimates events during the index hospitalization; it does not estimate death after discharge.

Subgroup fairness, decision thresholds, and safe use

Post hoc subgroup audits aligned with ISO/IEC TR 24027 identified threshold-dependent differences in prediction and error rates by recorded sex, age, and their intersection. These findings do not define an individual as advantaged or disadvantaged and do not establish a universal fairness criterion for this clinical context. The calculator therefore does not apply demographic parity constraints, group-specific thresholds, or automated allocation rules.

Decision-curve analysis showed positive internally cross-validated net benefit across threshold probabilities of 5%–50%, but those threshold probabilities represent relative weighting of false-positive and false-negative decisions. They are not recommended treatment, ICU-admission, triage, or resource-allocation thresholds.

Use the numerical probability as research information alongside clinical context. Do not automate treatment, discharge, escalation, or denial of care from this calculator.

Full-precision coefficients and methodological references

| Predictor | Center | β̂ death | β̂ discharge |
| --- | --- | --- | --- |

Cox DR. JRSS B. 1972;34:187–202. Efron B. JASA. 1977;72:557–565. Benichou J, Gail MH. Biometrics. 1990;46:813–826. Ozenne B et al. The R Journal. 2017;9:440–460. doi:10.32614/RJ-2017-062. Rice TW et al. Chest. 2007;132:410–417. doi:10.1378/chest.07-0617. Full definitions, estimation equations, coefficients, and baseline increments accompany the manuscript supplement. Model-based coefficient standard errors and covariance matrices are provided in the statistical supplement and model specification.

CSC45 · Multi-horizon research interface · 2026-10-02 · Research use only · Not a medical device or a substitute for clinical judgment.
