# Supplementary material for: Competing Risk Prediction of COVID-19 Hospital Mortality with Model Diagnostics and Exploratory Evaluation in Mexico
Source: Diagnostics (Basel). 2026 Oct 9;16(20):3267. doi: 10.3390/diagnostics16203267 (PMC13703144; doi:10.3390/diagnostics16203267)
Supplement: Supplementary file 1 [file diagnostics-16-03267-s001.zip › Supplement S3.pdf]

Supplement S3

ISO/IEC TR 24027-Aligned Bias and Fairness Assessment of the BCN CSC45 Mortality Model

Barcelona cohort: N=1,622; deaths by day 45=325; reference audit threshold=20%

Scope and status. This supplement reports a post-hoc subgroup bias and fairness audit aligned with ISO/IEC TR 24027:2021, Clauses 7.3-7.6. The audit evaluates equalized odds, equality of opportunity, demographic parity, and predictive equality using the frozen CSC45 45-day mortality probabilities. Statistical tests, effect sizes, threshold-sensitivity analyses, and an operational oxygenation-route audit are included. This is an ISO-aligned analytical assessment and not an ISO certification or conformity claim.

Key finding. At the 20% reference threshold, recorded-sex false-positive rates were similar (24.3% in females and 27.1% in males), whereas sensitivity was lower in females (65.0% vs 77.8%; equality-of-opportunity gap 12.8 percentage points, permutation p=0.0196, FDR q=0.0232). Age and sex-by-age analyses showed substantially larger threshold-dependent differences. These results identify subgroup operating-characteristic differences; they do not by themselves establish discriminatory or unethical bias.

S3.1. Methods

S3.1.1. Standards framework and fairness estimands

The audit followed the classification-fairness framework described in ISO/IEC TR 24027:2021 [1]. Equalized odds was evaluated through equality of both true-positive rate (TPR) and false-positive rate (FPR) across groups. Equality of opportunity was evaluated through equality of TPR among patients who died by day 45. Demographic parity assessed equality of the positive-classification rate irrespective of outcome, and predictive equality assessed equality of FPR among patients who did not die by day 45 [1-4].

ISO/IEC TR 24027 recommends that relevant group characteristics, fairness objectives, and an allowable margin of difference (delta) be specified for the intended use. No fairness delta was prespecified in the original CSC45 protocol. Accordingly, this audit reports subgroup rates, absolute rate gaps, standardized effect sizes, p values, and FDR-adjusted q values without retrospectively declaring the model fair, unfair, or ISO compliant.

S3.1.2. Cohort, outcome, and operating thresholds

The analysis used the unchanged 45-day mortality probabilities from the frozen CSC45 model in the 1,622-patient oxygenation-expanded Barcelona cohort. The positive condition was death during the index hospitalization on or before day 45. Patients discharged alive before day 45 or alive without death at day 45 were treated as condition-negative for this fixed-horizon classification audit.

A positive model decision was defined as predicted 45-day mortality risk >=20%. The 20% value was chosen as a reference audit operating point because it is close to the cohort event prevalence and lies within the previously examined decision-curve range; it is not proposed as a treatment, ICU-admission, or triage threshold. Threshold sensitivity was evaluated at 10%, 15%, 20%, 25%, 30%, and 40%.

S3.1.3. Group definitions

Primary demographic audits were performed for recorded sex (female vs male), age (<65, 65-74, and >=75 years), and the intersection of recorded sex and age. Age bands were selected for clinical interpretability and adequate cell sizes rather than optimized against the fairness results. Race/ethnicity, socioeconomic position, disability, and other potentially relevant protected attributes were not available and therefore could not be assessed.

Because ISO/IEC TR 24027 also identifies data-processing and missing-feature pathways as potential sources of bias, an additional non-demographic operational audit compared patients with directly measured versus saturation-derived P/F values. This analysis was interpreted as a measurement-route/case-mix sensitivity analysis, not as demographic fairness.

S3.1.4. Fairness metrics and effect sizes

For each criterion, the primary effect size was the maximum absolute difference across groups in the relevant rate. For demographic parity this was the max-min positive-prediction-rate gap; for equality of opportunity, the max-min TPR gap; for predictive equality, the max-min FPR gap; and for equalized odds, the larger of the TPR and FPR max-min gaps. Gaps are reported in percentage points. Cramer's V was additionally reported for demographic parity, equality of opportunity, and predictive equality as a standardized association effect size.

$$TPR_g = TP_g / (TP_g + FN_g)$$

$$FPR_g = FP_g / (FP_g + TN_g)$$

$$\text{Selection rate}_g = (TP_g + FP_g) / N_g$$

$$\text{Equality-of-opportunity gap} = \max_g(TPR_g) - \min_g(TPR_g)$$

$$\text{Predictive-equality gap} = \max_g(FPR_g) - \min_g(FPR_g)$$

$$\text{Demographic-parity gap} = \max_g(\text{Selection rate}_g) - \min_g(\text{Selection rate}_g)$$

$$\text{Equalized-odds gap} = \max\{\text{TPR gap}, \text{FPR gap}\}$$

S3.1.5. Statistical inference

P values were obtained using permutation tests because the fairness statistics are rate-gap statistics and some intersectional cells were small. For demographic parity, predicted-positive labels were permuted across all participants. For equality of opportunity and predictive equality, labels were permuted within the observed Y=1 and Y=0 strata, respectively. Equalized odds used outcome-stratified permutation and the maximum of the TPR and FPR gaps as the joint test statistic. The primary analyses used 20,000 permutations; threshold-sensitivity analyses used 5,000 permutations per test.

Benjamini-Hochberg false-discovery-rate (FDR) q values were calculated across the 12 primary demographic fairness tests. For post-hoc sex-within-age localization, Fisher's exact tests compared TPR and FPR between females and males within each age band. Statistical significance was interpreted as evidence against exact equality of the audited rates, not as proof of socially or clinically unacceptable bias; conversely, non-significance was not interpreted as proof of fairness.

S3.2. Results

S3.2.1. Subgroup operating characteristics at the 20% threshold

Among 597 females, 100 died by day 45; among 1,025 males, 225 died. The high-risk classification rate was 31.2% in females and 38.2% in males. TPR was 65.0% versus 77.8%, whereas FPR was 24.3% versus 27.1%. Mortality prevalence and model positivity rose strongly with age: the high-risk rate was 8.2% for patients <65 years, 34.9% for those aged 65-74 years, and 70.9% for those >=75 years; corresponding TPRs were 34.7%, 70.5%, and 86.2%, and FPRs were 6.1%, 24.3%, and 63.1%.

Audit dimension	Group	N	Deaths	Observed mortality	High-risk rate	TPR	FPR	PPV
Recorded sex	Female	597	100	16.8%	31.2%	65.0%	24.3%	34.9%
Recorded sex	Male	1025	225	22.0%	38.2%	77.8%	27.1%	44.6%
Age group	<65	673	49	7.3%	8.2%	34.7%	6.1%	30.9%
Age group	65–74	416	95	22.8%	34.9%	70.5%	24.3%	46.2%
Age group	≥75	533	181	34.0%	70.9%	86.2%	63.1%	41.3%

Table S2. Threshold-based performance by recorded sex and age group at the 20% predicted-risk threshold.

S3.2.2. Primary ISO/IEC TR 24027-aligned fairness tests

For recorded sex, demographic parity differed by 7.1 percentage points (Cramer's V=0.071; permutation p=0.0052; FDR q=0.0070). Equality of opportunity differed by 12.8 points because TPR was lower in females than males (Cramer's V=0.134; p=0.0196; q=0.0232). Predictive equality showed only a 2.8-point FPR gap and no statistical evidence of inequality (Cramer's V=0.031; p=0.276). The joint equalized-odds test was significant (max gap 12.8 points; p=0.0212; q=0.0232), driven primarily by the TPR difference.

All four age-group metrics showed large differences at the 20% threshold: demographic-parity gap 62.7 points (Cramer's V=0.561), equality-of-opportunity gap 51.5 points (V=0.407), predictive-equality gap 57.0 points (V=0.541), and equalized-odds max gap 57.0 points; all permutation p<0.0001 with FDR q<=0.0001. Intersectional sex-by-age differences were larger still, with gaps of 71.6 points for demographic parity, 54.0 for equality of opportunity, 66.0 for predictive equality, and 66.0 for equalized odds.

Audit dimension	Metric	Max-min gap (pp)	Cramer's V	Permutation p	FDR q
Recorded sex	Equalized odds	12.8	—	0.0212	0.0232
Recorded sex	Equality of opportunity	12.8	0.134	0.0196	0.0232
Recorded sex	Demographic parity	7.1	0.071	0.0052	0.0070
Recorded sex	Predictive equality	2.8	0.031	0.2757	0.2757
Age group	Equalized odds	57.0	—	<0.0001	<0.0001
Age group	Equality of opportunity	51.5	0.407	<0.0001	<0.0001
Age group	Demographic parity	62.7	0.561	<0.0001	<0.0001
Age group	Predictive equality	57.0	0.541	<0.0001	<0.0001
Recorded sex × age	Equalized odds	66.0	—	<0.0001	0.0002
Recorded sex × age	Equality of opportunity	54.0	0.485	0.0011	0.0017
Recorded sex × age	Demographic parity	71.6	0.581	<0.0001	<0.0001
Recorded sex × age	Predictive equality	66.0	0.553	<0.0001	<0.0001

Table S3. Primary fairness tests and effect sizes at the 20% audit threshold.

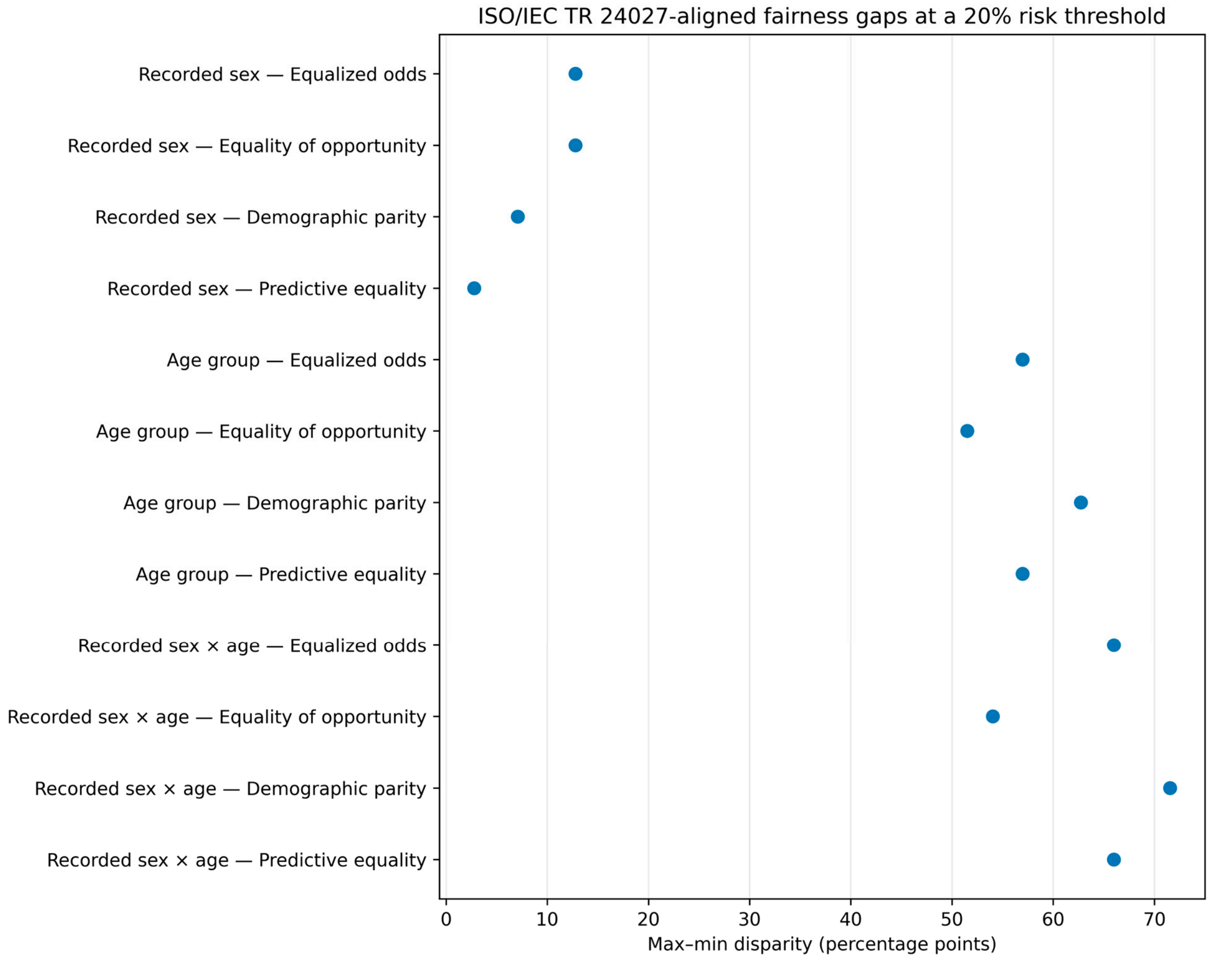

Figure S1. Max-min fairness gaps at the 20% reference threshold.

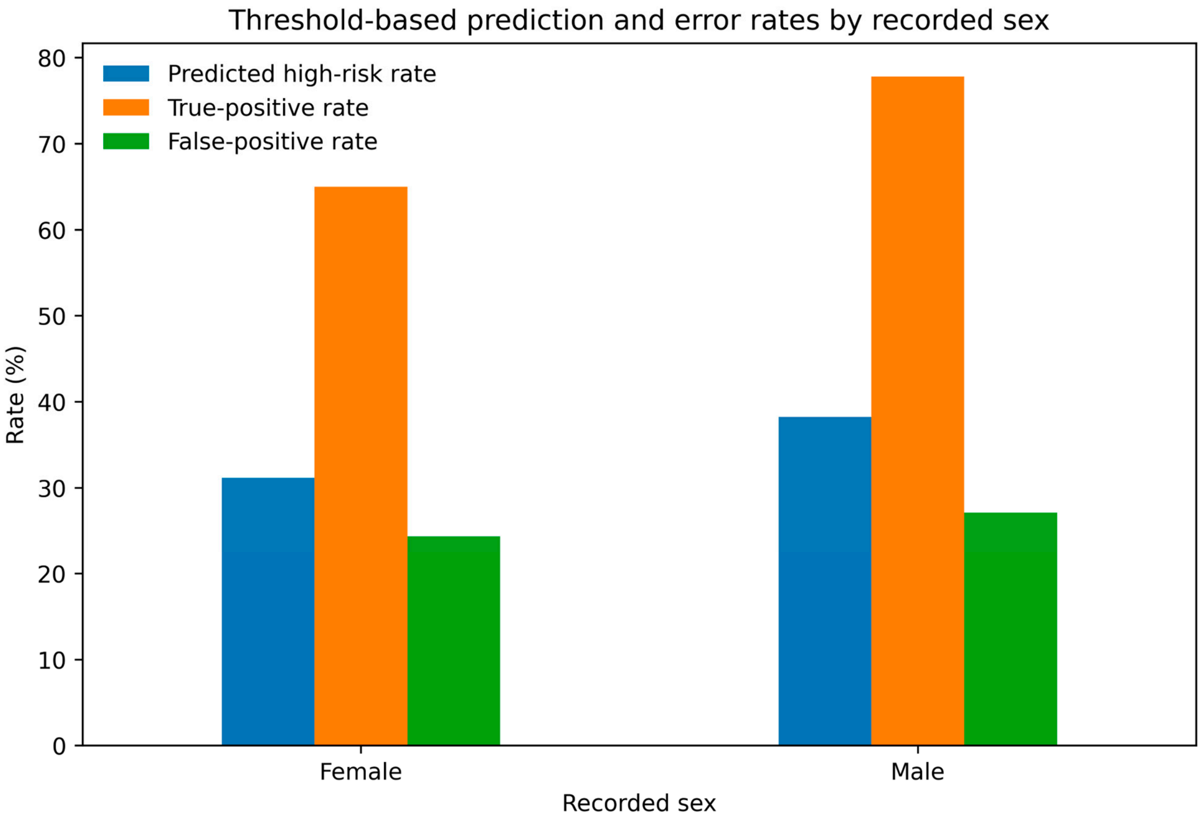

Figure S2. Positive-prediction rate, true-positive rate, and false-positive rate by recorded sex at the 20% threshold.

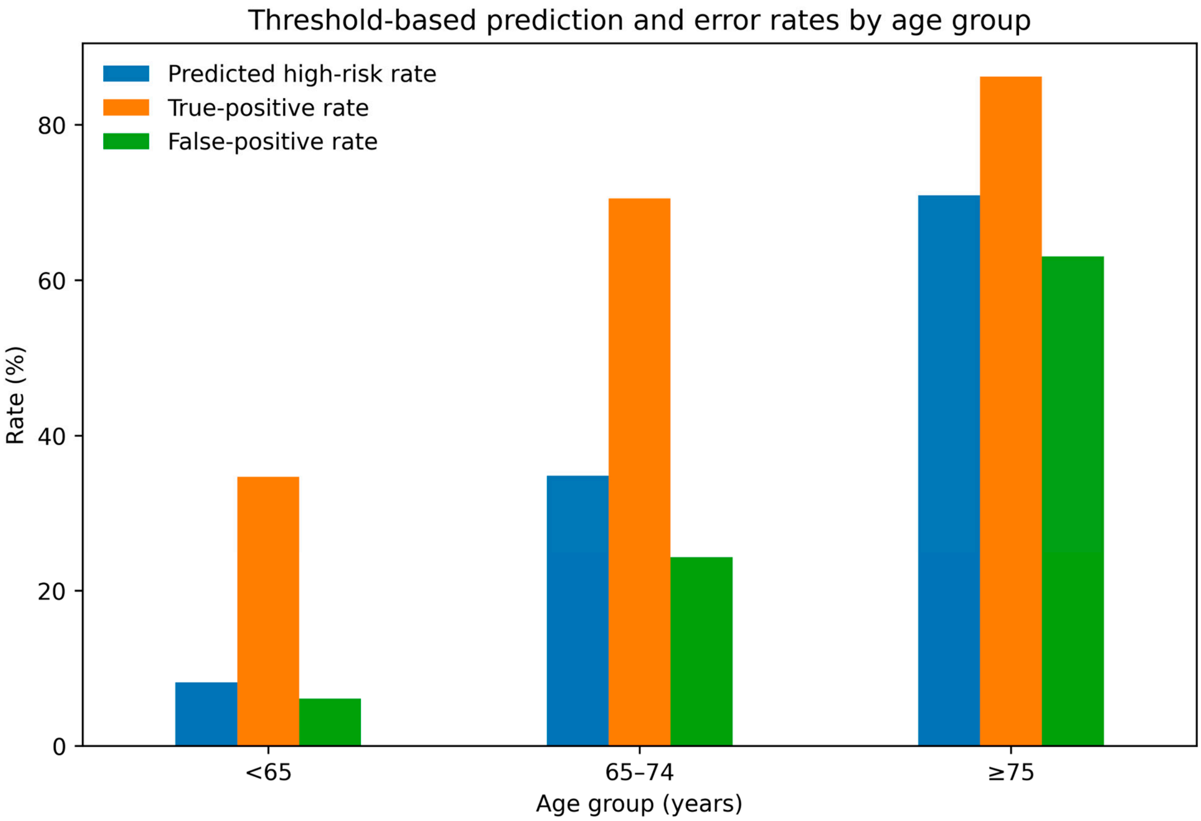

Figure S3. Positive-prediction rate, true-positive rate, and false-positive rate by age group at the 20% threshold.

S3.2.3. Threshold sensitivity

Sex-related findings were threshold dependent. The demographic-parity gap remained approximately 4.4-8.4 percentage points over thresholds from 10% to 40% and was statistically detectable at each tested threshold. In contrast, the sex TPR/equalized-odds gap was most evident at thresholds of 20%-30% (approximately 12.8-14.4 points) and was smaller and non-significant at 10%, 15%, and 40%. Predictive-equality gaps by sex remained small (approximately 1.4-5.2 points) and were not statistically significant across the tested thresholds. Age and intersectional gaps remained large across the full threshold range.

Threshold	Metric	Gap (pp)	Permutation p	FDR q
10%	Equalized odds	5.2	0.1622	0.1824
10%	Equality of opportunity	3.6	0.4091	0.4091
10%	Demographic parity	7.1	0.0058	0.0080
10%	Predictive equality	5.2	0.0738	0.0843
15%	Equalized odds	6.3	0.2034	0.2218
15%	Equality of opportunity	6.3	0.1884	0.2086
15%	Demographic parity	7.7	0.0018	0.0027
15%	Predictive equality	5.0	0.0734	0.0843
20%	Equalized odds	12.8	0.0226	0.0271
20%	Equality of opportunity	12.8	0.0216	0.0268
20%	Demographic parity	7.1	0.0044	0.0063
20%	Predictive equality	2.8	0.2667	0.2744
25%	Equalized odds	13.2	0.0208	0.0263
25%	Equality of opportunity	13.2	0.0192	0.0251
25%	Demographic parity	8.4	0.0012	0.0019
25%	Predictive equality	4.3	0.0576	0.0680
30%	Equalized odds	14.4	0.0208	0.0263
30%	Equality of opportunity	14.4	0.0188	0.0251
30%	Demographic parity	7.0	0.0026	0.0038
30%	Predictive equality	2.5	0.2444	0.2550
40%	Equalized odds	7.8	0.2310	0.2445
40%	Equality of opportunity	7.8	0.2222	0.2387
40%	Demographic parity	4.4	0.0226	0.0271
40%	Predictive equality	1.4	0.4027	0.4084

Table S4A. Threshold sensitivity: recorded sex.

Threshold	Metric	Gap (pp)	Permutation p	FDR q
10%	Equalized odds	73.6	0.0002	0.0004
10%	Equality of opportunity	39.7	0.0002	0.0004
10%	Demographic parity	73.0	0.0002	0.0004
10%	Predictive equality	73.6	0.0002	0.0004
15%	Equalized odds	68.5	0.0002	0.0004
15%	Equality of opportunity	46.4	0.0002	0.0004
15%	Demographic parity	71.1	0.0002	0.0004
15%	Predictive equality	68.5	0.0002	0.0004
20%	Equalized odds	57.0	0.0002	0.0004
20%	Equality of opportunity	51.5	0.0002	0.0004
20%	Demographic parity	62.7	0.0002	0.0004
20%	Predictive equality	57.0	0.0002	0.0004
25%	Equalized odds	51.5	0.0002	0.0004
25%	Equality of opportunity	51.5	0.0002	0.0004
25%	Demographic parity	56.5	0.0002	0.0004
25%	Predictive equality	48.8	0.0002	0.0004
30%	Equalized odds	56.8	0.0002	0.0004
30%	Equality of opportunity	56.8	0.0002	0.0004
30%	Demographic parity	49.0	0.0002	0.0004
30%	Predictive equality	38.6	0.0002	0.0004
40%	Equalized odds	45.8	0.0002	0.0004
40%	Equality of opportunity	45.8	0.0002	0.0004
40%	Demographic parity	32.6	0.0002	0.0004
40%	Predictive equality	21.3	0.0002	0.0004

Table S4B. Threshold sensitivity: age group.

Threshold	Metric	Gap (pp)	Permutation p	FDR q
10%	Equalized odds	85.8	0.0002	0.0004
10%	Equality of opportunity	54.5	0.0002	0.0004
10%	Demographic parity	84.2	0.0002	0.0004
10%	Predictive equality	85.8	0.0002	0.0004
15%	Equalized odds	80.0	0.0002	0.0004
15%	Equality of opportunity	57.8	0.0002	0.0004
15%	Demographic parity	81.9	0.0002	0.0004
15%	Predictive equality	80.0	0.0002	0.0004
20%	Equalized odds	66.0	0.0002	0.0004
20%	Equality of opportunity	54.0	0.0012	0.0019
20%	Demographic parity	71.6	0.0002	0.0004
20%	Predictive equality	66.0	0.0002	0.0004
25%	Equalized odds	59.0	0.0006	0.0010
25%	Equality of opportunity	56.4	0.0018	0.0027
25%	Demographic parity	65.9	0.0002	0.0004
25%	Predictive equality	59.0	0.0002	0.0004
30%	Equalized odds	60.8	0.0006	0.0010
30%	Equality of opportunity	60.8	0.0010	0.0016
30%	Demographic parity	57.2	0.0002	0.0004
30%	Predictive equality	45.5	0.0002	0.0004
40%	Equalized odds	50.8	0.0054	0.0076
40%	Equality of opportunity	50.8	0.0072	0.0098
40%	Demographic parity	37.8	0.0002	0.0004
40%	Predictive equality	24.6	0.0002	0.0004

Table S4C. Threshold sensitivity: recorded sex by age.

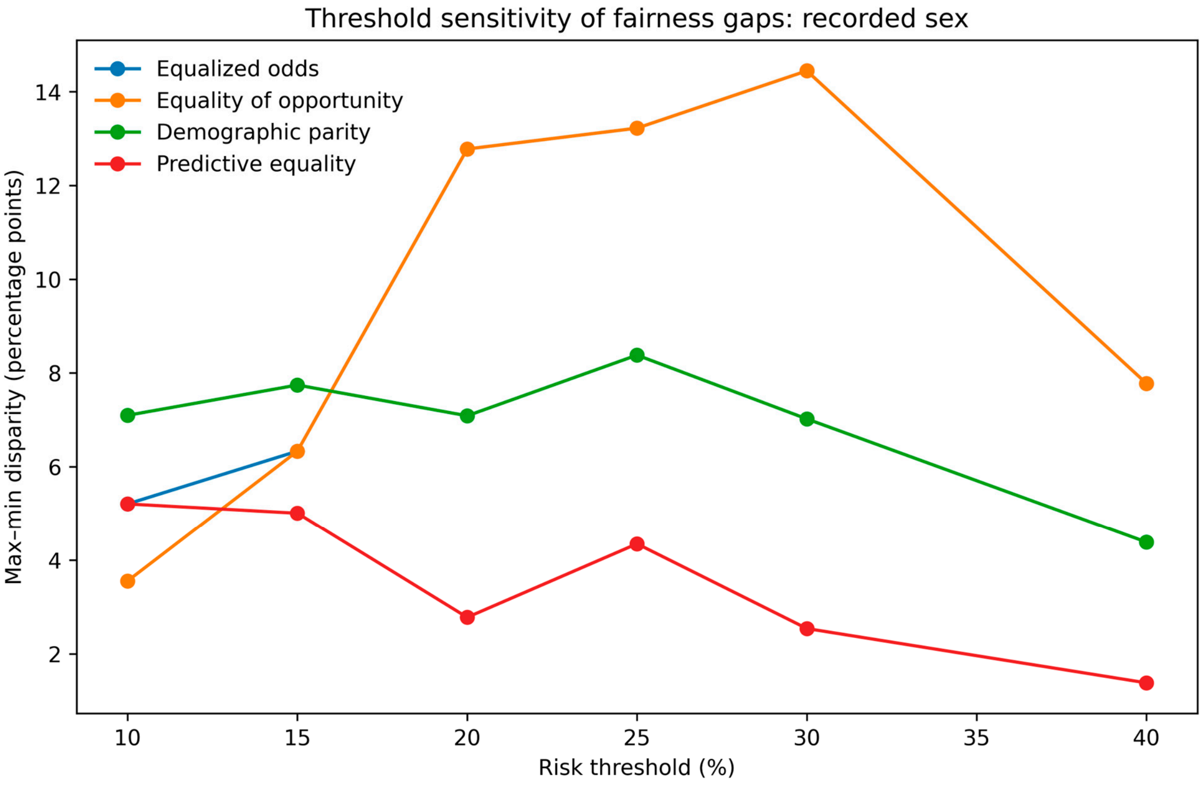

Figure S4. Threshold sensitivity of fairness gaps for recorded sex.

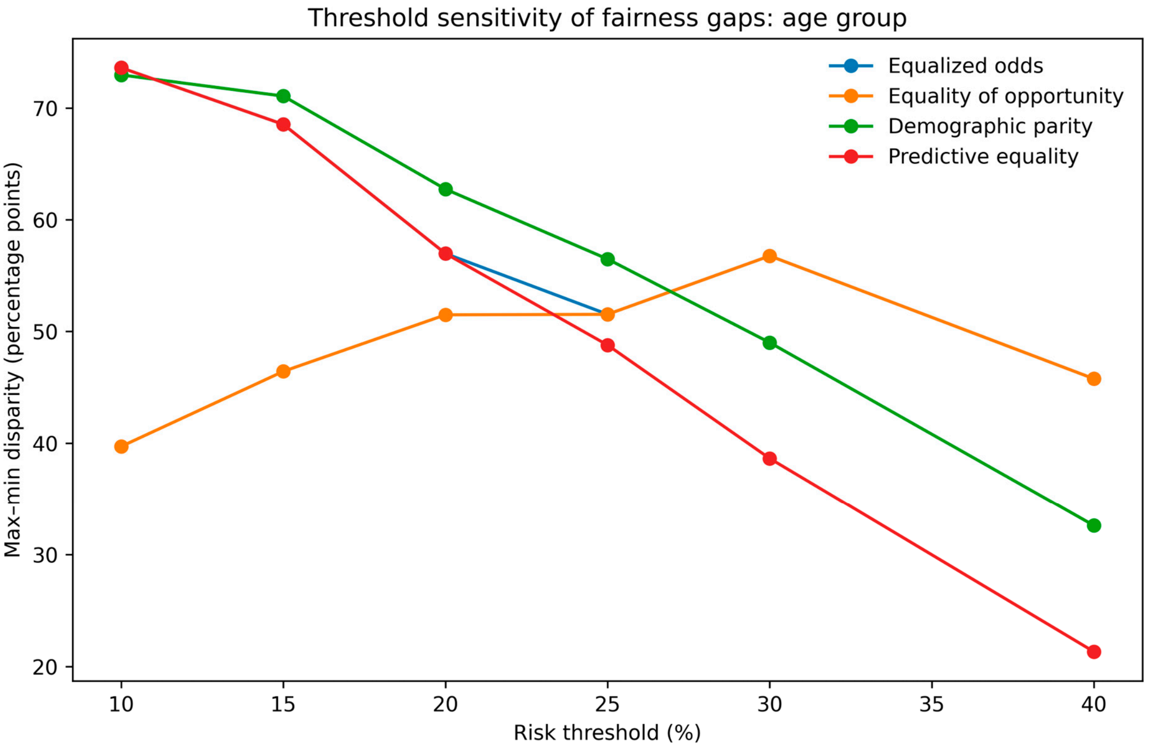

Figure S5. Threshold sensitivity of fairness gaps for age group.

S3.2.4. Intersectional localization

The intersectional audit showed that aggregate sex results can mask age-specific differences. The most prominent post-hoc localization occurred among patients aged 65-74 years: TPR was 37.0% for females and 83.8% for males, a 46.8-percentage-point difference (Fisher exact p=0.000026). In the same age band, FPR was 16.5% in females and 29.8% in males (difference 13.2 points; p=0.0080). Among patients <65 years, TPRs were similar (36.4% vs 34.2%; p=1.00), whereas among patients >=75 years TPRs were 82.3% and 88.2% (p=0.268). These localized comparisons were post hoc and should be externally validated.

Age group	Female N	Male N	Female TPR	Male TPR	TPR diff M-F	Fisher p TPR	Female FPR	Male FPR	FPR diff M-F	Fisher p FPR	Female high-risk	Male high-risk
<65	210	463	36.4%	34.2%	-2.2 pp	1.0000	3.5%	7.3%	3.8 pp	0.0733	5.2%	9.5%
65–74	160	256	37.0%	83.8%	46.8 pp	<0.0001	16.5%	29.8%	13.2 pp	0.0080	20.0%	44.1%
≥75	227	306	82.3%	88.2%	6.0 pp	0.2676	55.8%	69.5%	13.8 pp	0.0081	63.0%	76.8%

Table S5. Post-hoc sex comparisons within age groups at the 20% threshold.

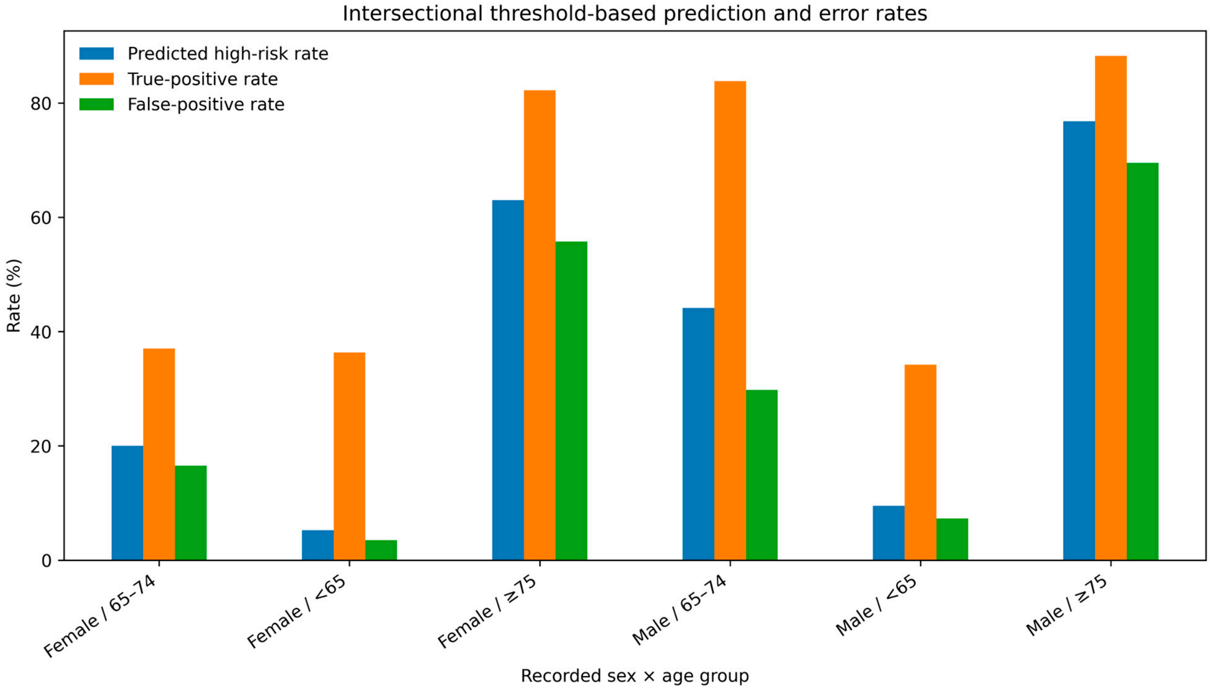

Figure S6. Intersectional prediction and error rates by recorded sex and age at the 20% threshold.

S3.2.5. Operational audit by oxygenation measurement route

This analysis addressed a possible data-processing pathway rather than demographic fairness. At the 20% reference threshold, the high-risk rate was 16.0% in the saturation-derived P/F group versus 46.8% in the directly measured P/F group. TPR was 44.9% versus 81.6%, and FPR was 12.1% versus 35.3%. The equalized-odds max gap was 36.7 points, equality-of-opportunity gap 36.7 points, demographic-parity-style gap 30.8 points, and predictive-equality gap 23.2 points; all permutation p<0.0001. Because the measured and derived P/F groups differed substantially in clinical severity, testing practice, and mortality, these differences do not identify the Rice derivation itself as the causal source of disparity.

Group	N	Deaths	Observed mortality	High-risk rate	TPR	FPR	PPV
Derived P/F	588	69	11.7%	16.0%	44.9%	12.1%	33.0%
Measured P/F	1034	256	24.8%	46.8%	81.6%	35.3%	43.2%

Table S6. Operating characteristics by oxygenation measurement route.

Metric	Gap (pp)	Cramer's V	Permutation p	TPR gap	FPR gap	Selection-rate gap
Equalized odds	36.7	—	<0.0001	36.7 pp	23.2 pp	—
Equality of opportunity	36.7	0.342	<0.0001	36.7 pp	—	—
Demographic parity	30.8	0.309	<0.0001	—	—	30.8 pp
Predictive equality	23.2	0.259	<0.0001	—	23.2 pp	—

Table S7. Operational fairness-style rate-gap tests by oxygenation measurement route.

S3.3. Interpretation and limitations

The audit identified three main findings. First, threshold-based performance was not fully invariant by recorded sex. At the 20% operating point, the FPR was similar between females and males, but sensitivity was approximately 13 percentage points lower in females. Consequently, predictive equality showed no statistical evidence of a sex difference, whereas equality of opportunity and the joint equalized-odds criterion did. The demographic-parity gap was smaller and should be interpreted alongside the lower observed mortality prevalence in females (16.8% vs 22.0%).

Second, demographic parity across age groups is not an appropriate stand-alone clinical fairness target in this setting because age is an intentionally included and clinically relevant prognostic factor and observed mortality differed strongly by age. Requiring equal high-risk classification rates across age bands would force the model to ignore clinically meaningful risk differences. Nevertheless, the large age differences in TPR and FPR are important quality-of-service findings because a single fixed threshold produces markedly different error profiles across age groups.

Third, the intersectional analysis showed that aggregate sex results can conceal subgroup-specific behavior. The 65-74-year female subgroup had substantially lower TPR than males of the same age band, but this was a post-hoc localization with only 27 female deaths. It should therefore be independently validated rather than used to tune a sex-specific threshold in the same data.

Statistical significance is not equivalent to unfairness, and absence of significance is not proof of fairness. No prospective fairness acceptance delta was defined in the original study. Future clinical deployment would require prespecification of clinically and ethically justified acceptance margins, external subgroup validation, and monitoring over time. Race/ethnicity, socioeconomic position, disability, and several other potentially relevant characteristics were unavailable, so this audit cannot establish fairness across the broader target population.

The 20% threshold is an audit operating point rather than a treatment threshold. Threshold-sensitivity analysis was therefore included to show how conclusions depend on the operating point. The measured-versus-derived P/F analysis should likewise be interpreted cautiously: the observed differences may arise from disease severity, arterial-testing practice, and other case-mix factors rather than the derivation equation itself.

S3.4. Reproducibility notes

All demographic primary analyses used 20,000 Monte Carlo permutations and Benjamini-Hochberg FDR control across the 12 primary tests. Threshold-sensitivity analyses used 5,000 permutations per test. The smallest attainable Monte Carlo p values are therefore determined by the number of permutations; values reported as <0.0001 in the primary audit correspond to the stated simulation resolution rather than an exact zero.

The audit was performed on fixed model predictions and does not include model refitting within demographic subgroups. It therefore assesses subgroup operating characteristics of the frozen CSC45 model at prespecified thresholds rather than estimating separate subgroup-specific prognostic models.

References

1. ISO/IEC. ISO/IEC TR 24027:2021. Information technology — Artificial intelligence (AI) — Bias in AI systems and AI aided decision making. Geneva: International Organization for Standardization; 2021.

2. Hardt M, Price E, Srebro N. Equality of Opportunity in Supervised Learning. Advances in Neural Information Processing Systems. 2016;29:3315-3323.

3. Verma S, Rubin J. Fairness Definitions Explained. Proceedings of the 2018 IEEE/ACM International Workshop on Software Fairness (FairWare '18). 2018:1-7. doi:10.1145/3194770.3194776.

4. Mitchell S, Potash E, Barocas S, D'Amour A, Lum K. Prediction-Based Decisions and Fairness: A Catalogue of Choices, Assumptions, and Definitions. arXiv:1811.07867.

5. Saleiro P, Kuester B, Hinkson L, London J, Stevens A, Anisfeld A, Rodolfa KT, Ghani R. Aequitas: A Bias and Fairness Audit Toolkit. arXiv:1811.05577.
