# Supplementary material for: Competing Risk Prediction of COVID-19 Hospital Mortality with Model Diagnostics and Exploratory Evaluation in Mexico
Source: Diagnostics (Basel). 2026 Oct 9;16(20):3267. doi: 10.3390/diagnostics16203267 (PMC13703144; doi:10.3390/diagnostics16203267)
Supplement: Supplementary file 1 [file diagnostics-16-03267-s001.zip › Supplement S5.pdf]

Supplement S5. TRIPOD+AI Checklist — Resolved Reporting Version

Item	Topic	Requirement (paraphrased)	Page / section	Status
1	Title	Identify prediction-model study, target population, and outcome.	p.1, Title	Reported
2	Abstract	Structured summary of objective, data, participants, predictors, outcome, methods, results, and conclusions.	p.1, Abstract	
3a	Background	Explain the clinical context and rationale, including existing models.	pp.2–3, Introduction	Reported
3b	Target population / intended use / users	Describe target population, intended purpose in the care pathway, and intended users.	Introduction + Section 2.11	Reported
3c	Health inequalities	Describe known health inequalities between relevant sociodemographic groups.	Introduction + Section 4 + Supplement S3	Reported
4	Objectives	State whether models are developed and/or evaluated.	p.3, end of Introduction	Reported
5a	Data sources	Describe development/evaluation data sources and rationale.	Sections 2.1, 2.8; Table 2	Reported
5b	Dates	Report accrual and follow-up dates.	Sections 2.1, 2.9; Table 2	Reported
6a	Setting	Describe healthcare setting, centres, and geography.	Sections 2.1–2.2; Table 2	Reported
6b	Eligibility	Describe eligibility criteria.	Section 2.1; Section 2.8	Reported
6c	Treatments	Report relevant treatments and how handled in development/evaluation.	Section 2.1 / 2.9	Reported
7	Data preparation	Describe preprocessing, harmonisation, derivation, and quality checks.	Sections 2.4–2.8; Supplements S2–S4	Reported
8a	Outcome	Define outcome, timing, rationale, and prediction horizon.	Section 2.3	Reported
8b	Outcome assessor qualifications	Describe assessors if outcome requires subjective interpretation.	N/A	N/A
8c	Outcome blinding	Describe blinding of outcome assessment to predictors if relevant.	N/A	N/A
9a	Predictor selection	Describe predictor selection/preselection.	Section 2.4	Reported
9b	Predictor definitions	Define predictors, timing, units, and measurement.	Section 2.4; Supplement S4	Reported
9c	Predictor assessor qualifications	Describe assessor qualifications for subjective predictors if relevant.	N/A	N/A
10	Sample size	Explain how sample size was determined for development/evaluation.	Section 2.1; Results Sections 3.1, 3.5	Reported
11	Missing data	Describe handling of missing predictor/outcome data.	Sections 2.4–2.5; Section 3.4	Reported
12a	Use/partitioning of data	Describe development/evaluation use and data partitioning.	Section 2.7	Reported
12b	Predictor handling	Describe functional form, transformations, scaling, standardisation.	Sections 2.4, 2.6	Reported
12c	Model building / internal validation	Specify model types, rationale, fitting/tuning, and internal validation.	Sections 2.6–2.7	Reported
12d	Cluster heterogeneity	Describe handling of clustering where applicable.	Section 2.9	N/A / complementary
12e	Performance assessment	Specify discrimination, calibration, clinical utility, plots, comparisons.	Sections 2.7, 2.9–2.10	Reported
12f	Model updating	Describe recalibration/updating	Sections 2.8, 3.5	N/A

		after evaluation.		
12g	Prediction calculation in evaluation data	Describe how evaluation predictions were generated.	Section 2.8	Reported
13	Class imbalance	If imbalance methods used, describe them and recalibration.	Methods	N/A
14	Fairness	Describe fairness assessment or mitigation methods and rationale.	Section 4; Figure 11; Supplement S3	Reported
15	Model output	Specify model output and rationale for thresholds.	Sections 2.6, 2.10, 3.8	Reported
16	Development vs evaluation differences	Identify differences in setting, eligibility, outcome, and predictors.	Sections 2.8, 3.5, 4	Reported
17	Ethics / consent	Name ethics committees and consent/waiver.	Sections 2.2; declarations	Reported
18a	Funding	Give funding source and funder role.	Funding statement	Reported
18b	Conflicts of interest	Declare conflicts/financial disclosures.	Declarations	Reported
18c	Protocol	State where protocol can be accessed or that none was prepared.	Open Science statement	Reported
18d	Registration	Give registry details or state study not registered.	Open Science statement	Reported
18e	Data sharing	Describe study-data availability.	Data Availability Statement (corrected)	Reported
18f	Code sharing	Describe analytical-code availability.	Section 2.11 + Data Availability	Reported
19	Patient/public involvement	Describe PPI or state none.	Declarations / PPI statement	Reported
20a	Participant flow	Report participant flow, outcomes, follow-up.	Section 3.1; Table 3	Reported
20b	Participant characteristics	Report overall/source-specific characteristics, predictors, treatments, events, missingness.	Section 3.1; Table 3; Section 3.6	Reported
20c	Evaluation vs development distributions	Compare important predictor/outcome distributions between development and evaluation data.	Sections 2.8, 3.5, 4	Reported
21	Numbers in each analysis	Report participants/outcome events for each analysis.	Results 3.1–3.7	Reported
22	Full model specification	Provide enough detail for independent calculation/evaluation.	Sections 2.6, 3.8; Supplements S2/S4	Reported
23a	Model performance	Report performance with uncertainty and key subgroup results.	Results 3.3–3.7; Supplement S3	Reported
23b	Cluster heterogeneity	Report cluster heterogeneity if examined.	N/A	N/A
24	Model updating results	Report updated model/performance if updating performed.	N/A	N/A
25	Interpretation	Interpret findings, including fairness, without overstatement.	Section 4	Reported
26	Limitations	Discuss limitations affecting bias, uncertainty, and generalisability.	Section 4	Reported
27a	Unavailable/poor-quality inputs	Describe handling in intended use.	Sections 2.4, 3.8, 4	Reported
27b	User interaction/expertise	Describe required user interaction and expertise.	Section 2.11 / 3.8	Reported
27c	Next research steps	Discuss future validation, implementation, and generalisability.	Sections 4–5	Reported

TRIPOD+AI reference: Collins GS, Moons KGM, Dhiman P, et al. TRIPOD+AI statement: updated guidance for reporting clinical prediction models that use regression or machine learning methods. BMJ. 2024;385:e078378.
