# Supplementary material for: Competing Risk Prediction of COVID-19 Hospital Mortality with Model Diagnostics and Exploratory Evaluation in Mexico
Source: Diagnostics (Basel). 2026 Oct 9;16(20):3267. doi: 10.3390/diagnostics16203267 (PMC13703144; doi:10.3390/diagnostics16203267)
Supplement: Supplementary file 1 [file diagnostics-16-03267-s001.zip › Supplementary Note S1.pdf]

Supplementary Note S1. Artificial Intelligence and Machine Learning in Clinical Prognosis: Relevance to the CSC45 Study

S1.1. Scope and terminology

Artificial intelligence (AI) and machine learning (ML) are increasingly used in healthcare to identify prognostic patterns from routinely collected electronic health record (EHR) data, including demographics, vital signs, oxygenation, laboratory results, comorbidities, and organ-support variables. In hospitalized and intensive-care populations, applications relevant to the present study include mortality prediction, deterioration detection, discharge/readmission support, time-to-event modeling, and dynamic risk stratification. Across the literature summarized in the accompanying evidence review, routine EHR variables frequently provide useful prognostic discrimination, longitudinal measurements can improve performance over static admission-only models, and boosted ensembles often outperform conventional regression in discrimination, although simpler models can remain competitive and easier to calibrate, explain, and maintain. The term AI is broad. The present CSC45 model is not a black-box AI system; it is an interpretable cause-specific Cox competing-risk model. Nevertheless, it addresses the same clinical-prediction problem as many contemporary ML systems and shares the same methodological challenges: predictor availability, outcome definition, missing data, transparency, calibration, competing events, external and temporal validation, decision utility, and subgroup performance.

S1.2. AI/ML applications relevant to hospitalized and ICU prognosis

Routine EHR-based mortality and deterioration prediction

Routine structured data are often sufficient for useful inpatient and ICU prognosis. Common predictors include age, respiratory status, oxygenation, comorbidity, renal and inflammatory markers, and other admission variables. Longitudinal models that update risk with repeated measurements often outperform static admission-only scores because inpatient physiology evolves rapidly.

Interpretable and explainable ML

SHAP, LIME, partial-dependence analyses, sparse models, and rule-based representations are widely used to expose clinically plausible drivers of prediction. However, explanation quality and bedside usefulness are distinct; transparency has advanced faster than evidence that explanations improve clinical outcomes or decision quality.

Survival, competing risks, and time-to-event prediction

Hospital prognosis concerns not only whether an event occurs but when it occurs and which competing events intervene. Cox, discrete-time hazard, random-survival-forest, and neural survival approaches have been used, but inconsistent handling of discharge, censoring, and missingness can change the estimand and limit comparability.

Causal and policy-learning decision support

Causal ML extends prediction toward outcomes under alternative actions. The reviewer-suggested ICU discharge study combined inverse-propensity weighting and doubly robust estimation with gradient-boosted trees and logistic regression to learn discharge policies from MIMIC-IV and eICU data. Such analyses target a different estimand from ordinary prognosis and remain dependent on confounding and modeling assumptions.

External validation, temporal validation, and dataset shift

External validation can reveal lower discrimination and substantial calibration error even when derivation performance is strong. Temporal validation likewise shows that discrimination may remain acceptable while calibration drifts as treatment, admission thresholds, variants, immunity, and practice patterns change. These findings support explicit recalibration and monitoring.

Supplementary Table S1.A. Representative AI/ML and clinical-prediction studies relevant to the present work

Study	Setting / data	Approach	Outcome / use	Reported performance / validation	Relevance to CSC45
Churpek et al., 2016	Multicenter hospital wards	Multiple ML methods vs conventional regression	Clinical deterioration	Multicenter comparison of ML and conventional approaches	Routine EHR data and explicit model comparison.
Ebell et al., 2021	Six US health systems; hospitalized COVID-19	COVID-NoLab / COVID-SimpleLab scores	COVID-19 prognosis	AUROC ~0.803 without labs; ~0.833 with routine labs/comorbidity	Useful prediction from routine admission data.
Knight et al., 2020	ISARIC hospitalized COVID-19 cohort	4C Mortality Score	In-hospital mortality	AUROC ~0.79 derivation and ~0.77 validation; calibration reported	Parsimonious risk stratification and validation/calibration.
Marcolino et al., 2021	Hospitalized COVID-19; Brazilian and Spanish validation	ABC2-SPH clinical score	In-hospital mortality	AUROC 0.844 derivation, 0.859 Brazilian validation, 0.894 Spanish validation	Transport of a routine-variable score across settings.
He et al., 2022	Multicenter hospitalized COVID-19 EHR	Simplified ML algorithms	Mortality, ICU admission, respiratory failure, ventilation	Test AUC ~0.89; prospective later-pandemic AUC ~0.85	Longitudinal/multicenter ML and later-period validation.
Kamran et al., 2022	COVID-19; one development site and 12 external hospitals	ML deterioration prediction	Clinical deterioration	External AUROC ~0.77-0.84; later-quarter ~0.73-0.83	Geographic and temporal transportability.
Hu et al., 2024	ICU mortality derivation and validation	Explainable AI / feature attribution	ICU mortality risk	Derivation/validation with explainability reporting	Transparent AI and clinically interpretable feature attribution.
Momenzadeh et al., 2026	MIMIC-IV 45,281 ICU stays; eICU 38,967 stays	Causal ML; IPW; doubly robust estimation; GBDT + logistic regression	ICU discharge policy / 30-day readmission	AUROC 0.847 and 0.821; counterfactual policy evaluation	Reviewer-suggested policy-oriented EHR decision support.
Pourmahmoudi et al., 2026	Iranian adults; 3,203 records; held-out test n=641	Leakage-free interpretable ML; 12 classifiers; SHAP	Anemia risk without diagnosis-defining indices	Logistic regression AUC 0.8625 (95% CI 0.8168-0.9053)	Reviewer-suggested transparent, leakage-aware prediction.
de Jong et al., 2022	IPD external validation across 27 clusters / 18 countries	Validation of COVID-19 mortality models	COVID-19 mortality	4C Mortality Score pooled C-statistic ~0.80; heterogeneity across settings	External validation and calibration are essential.
Rockenschaub et al., 2025	Systematic review/meta-analysis of ICU AI risk scores	AI/ML external-validation review	ICU outcomes	Only 14.7% of 572 studies externally validated; mean AUROC drop ~0.037	Quantifies the external-validation gap in clinical AI.
Minne et al., 2012	Time-ordered ICU data	Customized SAPS-II monitoring/recalibration	ICU mortality / quality assessment	Discrimination stable while calibration/SMR drifted until recalibration	Parallels CSC45 temporal calibration drift.
Current CSC45 study	Barcelona development plus Mexico evaluation	Interpretable cause-specific Cox competing-risk model	45-day in-hospital mortality with discharge alive competing	Internal, temporal, missing-data, oxygenation-route, Fine-Gray, DCA and subgroup analyses	Transparent survival model within broader AI/ML prognosis landscape.

Note: Performance values are study-specific and are not directly comparable because populations, endpoints, prevalence, predictors, validation designs, and time horizons differ.

Supplementary Table S1.B. Methodological themes from AI/ML literature and their relevance to CSC45

Theme	Typical AI/ML approach	Potential advantage	Key methodological risk	How addressed or interpreted in CSC45
Routine EHR prognosis	Logistic/regularized regression, tree ensembles, neural networks	Scalable prediction from routine data	Missingness, data quality, leakage, site-specific coding	Six admission predictors; missing-data and oxygenation-route sensitivity analyses.
Longitudinal prediction	RNN/LSTM, trajectory models, repeated-measure ensembles	Captures evolving physiology	Information leakage and timing ambiguity	CSC45 intentionally uses admission/early measurements.
Explainability	SHAP, LIME, PDP/ICE, sparse models	Transparency and feature attribution	Explanation does not prove causality or utility	Coefficients/HRs directly interpretable; no claim that interpretability proves benefit.
Survival / competing risks	Cox, discrete-time survival, RSF, DeepSurv, Fine-Gray	Explicit time-to-event modeling	Censoring/discharge rules alter estimand	Death/discharge modeled separately; Fine-Gray sensitivity performed.
Causal decision support	Propensity weighting, doubly robust estimation, policy learning	Targets consequences of alternative actions	Unmeasured confounding; counterfactual assumptions	CSC45 is prognostic, not causal; no treatment-effect claim.
External validation	Independent-site validation	Tests transportability	Case-mix and workflow shift	Mexico evaluation interpreted cautiously with endpoint/horizon limitations.
Temporal validation	Later-period holdout, drift monitoring	Tests stability as practice changes	Calibration drift despite stable AUC	Wave-based and chronological temporal validation; later-wave drift reported.
Clinical utility	Decision-curve analysis / impact studies	Connects predictions to decision consequences	Net benefit does not prove improved outcomes	DCA added; thresholds not presented as treatment/ICU recommendations.
Bias / subgroup performance	Fairness metrics, subgroup calibration/error analysis	Identifies differential performance	Metric choice and threshold dependence	ISO/IEC TR 24027-aligned subgroup analysis reported diagnostically.

Data leakage, outcome circularity, and methodological safeguards

The reviewer-suggested anemia study provides a useful methodological example. After removing diagnosis-defining hematologic indices and rebuilding feature selection, oversampling, and tuning inside a leakage-free training pipeline, the corrected model retained useful discrimination but no longer showed near-perfect performance. This illustrates why clinically meaningful prediction requires strict separation of predictors from the outcome definition and careful leakage control.

Supplementary References

1. Parchure P, Joshi H, Dharmarajan K, et al. Development and validation of a machine learning-based prediction model for near-term in-hospital mortality among patients with COVID-19. BMJ Support Palliat Care. 2020. doi:10.1136/bmjspcare-2020-002602.

2. Churpek MM, Yuen TC, Winslow C, et al. Multicenter comparison of machine learning methods and conventional regression for predicting clinical deterioration on the wards. Crit Care Med. 2016. doi:10.1097/CCM.0000000000001571.

3. Ebell MH, Cai X, Lennon R, et al. Development and validation of the COVID-NoLab and COVID-SimpleLab risk scores for prognosis in 6 US health systems. J Am Board Fam Med. 2021. doi:10.3122/JABFM.2021.S1.200464.

4. Knight SR, Ho A, Pius R, et al. Risk stratification of patients admitted to hospital with COVID-19 using the ISARIC WHO Clinical Characterisation Protocol: development and validation of the 4C Mortality Score. BMJ. 2020;370:m3339. doi:10.1136/bmj.m3339.

5. Marcolino MS, Pires MC, Ramos LEF, et al. ABC2-SPH risk score for in-hospital mortality in COVID-19 patients: development, external validation and comparison with other available scores. Int J Infect Dis. 2021;110:281-308. doi:10.1016/j.ijid.2021.07.049.

6. He F, Page JH, Weinberg KR, Mishra A. The development and validation of simplified machine learning algorithms to predict prognosis of hospitalized patients with COVID-19: multicenter, retrospective study. J Med Internet Res. 2022. doi:10.2196/31549.

7. Kamran F, Tang S, Otles E, et al. Early identification of patients admitted to hospital for COVID-19 at risk of clinical deterioration: model development and multisite external validation study. BMJ. 2022. doi:10.1136/bmj-2021-068576.

8. Aghakhani A, Shoshtarian Malak J, Karimi Z, et al. Predicting the COVID-19 mortality among Iranian patients using tree-based models: a cross-sectional study. Health Sci Rep. 2023. doi:10.1002/hsr2.1279.

9. Hu C, Gao C, Li T, et al. Explainable artificial intelligence model for mortality risk prediction in the intensive care unit: a derivation and validation study. Postgrad Med J. 2024. doi:10.1093/postmj/qgad144.

10. Ge W, Huh JW, Park YR, et al. An interpretable ICU mortality prediction model based on logistic regression and recurrent neural networks with LSTM units. AMIA Annu Symp Proc. 2018.

11. Momenzadeh M, Ghaderzadeh M, Oshaghi A, et al. A causal machine learning framework for ICU discharge decision support using electronic health records. Sci Rep. 2026. doi:10.1038/s41598-026-70894-8.

12. Pourmahmoudi A, Malekzadeh J, Noormohammadi A, et al. Predicting anemia risk in Iranian adults without diagnostic blood indices: an interpretable machine learning approach integrating dietary, clinical, and non-diagnostic laboratory patterns. Sci Rep. 2026. doi:10.1038/s41598-026-72618-4.

13. de Jong VMT, Rousset RZ, Antonio-Villa NE, et al. Clinical prediction models for mortality in patients with COVID-19: external validation and individual participant data meta-analysis. BMJ. 2022. doi:10.1136/bmj-2021-069881.

14. Gupta RK, Marks M, Samuels THA, et al. Systematic evaluation and external validation of 22 prognostic models among hospitalised adults with COVID-19: an observational cohort study. Eur Respir J. 2020. doi:10.1183/13993003.03498-2020.

15. Rockenschaub P, Akay EM, Carlisle BG, et al. External validation of AI-based scoring systems in the ICU: a systematic review and meta-analysis. BMC Med Inform Decis Mak. 2025. doi:10.1186/s12911-024-02830-7.

16. Minne L, Eslami S, de Keizer N, et al. Effect of changes over time in the performance of a customized SAPS-II model on the quality of care assessment. Intensive Care Med. 2012. doi:10.1007/s00134-011-2390-2.

17. Kim GYE, Corbin CK, Grolleau F, et al. Monitoring strategies for continuous evaluation of deployed clinical prediction models. J Biomed Inform. 2025. doi:10.1016/j.jbi.2025.104854.

18. Wynants L, Van Calster B, Collins GS, et al. Prediction models for diagnosis and prognosis of COVID-19: systematic review and critical appraisal. BMJ. 2020;369:m1328. doi:10.1136/bmj.m1328.

19. Liu L, Song W, Patil N, et al. Predicting COVID-19 severity: challenges in reproducibility and deployment of machine learning methods. Int J Med Inform. 2023. doi:10.1016/j.ijmedinf.2023.105210.

20. Shakibfar S, Nyberg F, Li H, et al. Artificial intelligence-driven prediction of COVID-19-related hospitalization and death: a systematic review. Front Public Health. 2023. doi:10.3389/fpubh.2023.1183725.
