# Supplementary material for: Competing Risk Prediction of COVID-19 Hospital Mortality with Model Diagnostics and Exploratory Evaluation in Mexico
Source: Diagnostics (Basel). 2026 Oct 9;16(20):3267. doi: 10.3390/diagnostics16203267 (PMC13703144; doi:10.3390/diagnostics16203267)
Supplement: Supplementary file 1 [file diagnostics-16-03267-s001.zip › Supplement File S2.zip!/figures/PH_Death_all_residuals.pdf]

# Death model — proportional hazards diagnostics

Barcelona n = 1,622 | 346 cause-specific events | Full-hospitalization fit
Global log(1 + time) score test: χ²(6) = 49.881; p = 5.0e-09

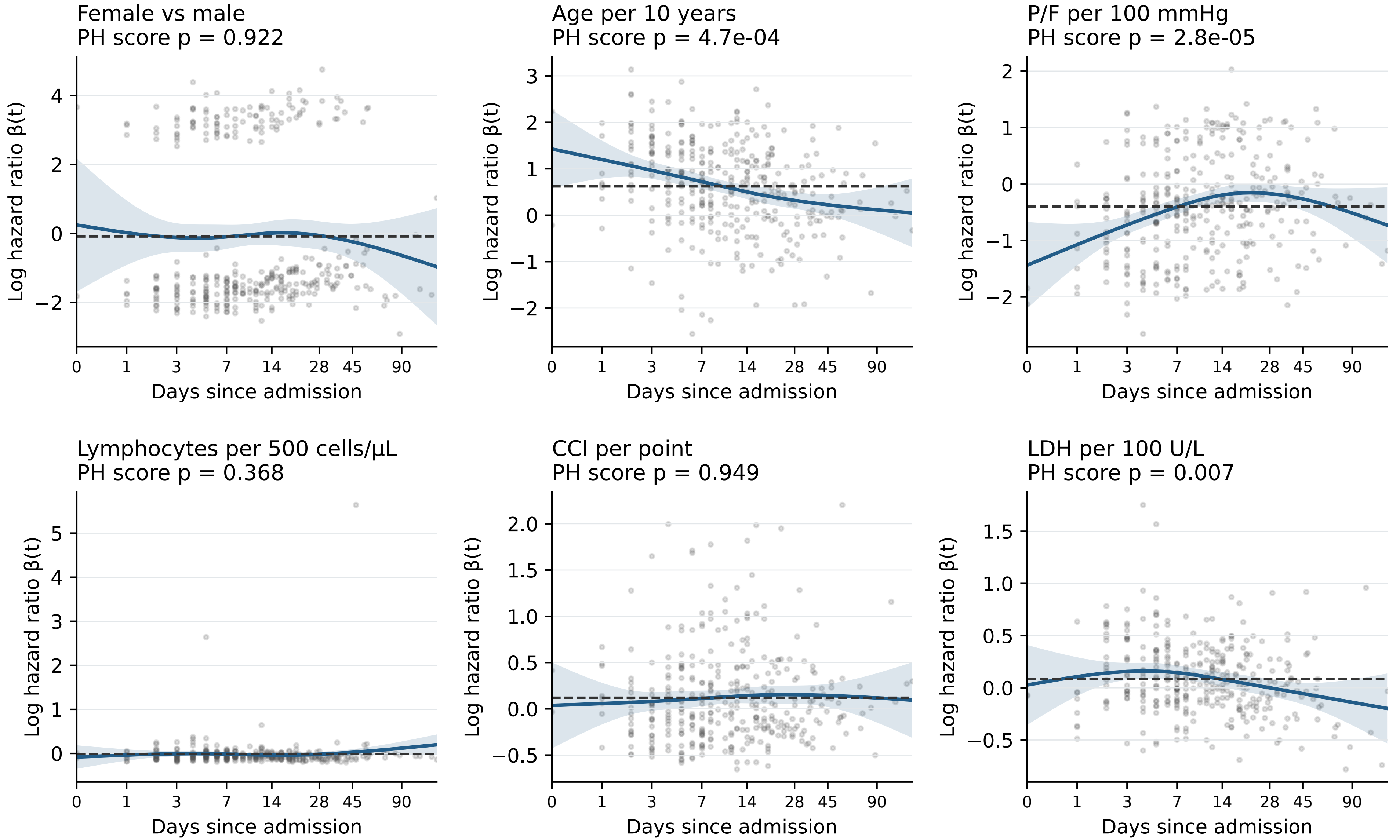


Curves: natural-cubic-spline smooths (4 df) of scaled Efron Schoenfeld residuals + fitted coefficients.
Shading: approximate ±2 SE; dashed lines: constant fitted coefficients. Time spacing uses log(1 + days).
All event residuals are shown; no residual values are trimmed.
