# Supplementary material for: Competing Risk Prediction of COVID-19 Hospital Mortality with Model Diagnostics and Exploratory Evaluation in Mexico
Source: Diagnostics (Basel). 2026 Oct 9;16(20):3267. doi: 10.3390/diagnostics16203267 (PMC13703144; doi:10.3390/diagnostics16203267)
Supplement: Supplementary file 1 [file diagnostics-16-03267-s001.zip › Supplement File S2.zip!/figures/PH_risk_set_support.pdf]

Risk-set support for the PH diagnostics

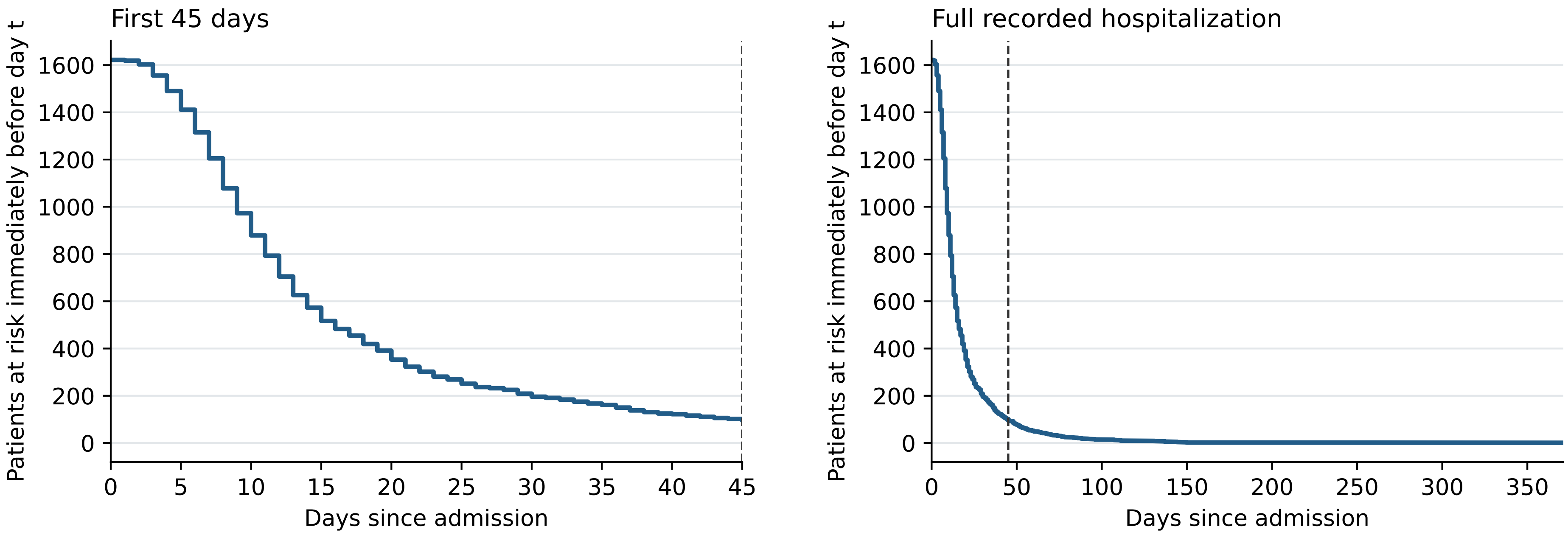

At risk on days 0, 7, 14, 28, 45, 90, 180: 1622, 1205, 573, 225, 95, 19, 2.
A shared risk set is used for both cause-specific models; either terminal event removes a patient.
